# Physical frailty, genetic predisposition, and risk of incident degenerative aortic valve stenosis: A prospective cohort study

**DOI:** 10.1016/j.jnha.2025.100648

**Published:** 2025-08-06

**Authors:** Bingbing Su, Piaopiao Zhu, Chaolei Chen, Zhanhao Su, Tiemei Shen

**Affiliations:** a Department of Nursing, Guangdong Provincial People’s Hospital (Guangdong Academy of Medical Sciences), Southern Medical University, Guangzhou, China; b Department of Cardiology, Guangdong Cardiovascular Institute, Guangdong Provincial People’s Hospital (Guangdong Academy of Medical Sciences), Southern Medical University, Guangzhou, China; c Department of Cardiovascular Surgery, Guangdong Cardiovascular Institute, Guangdong Provincial People's Hospital (Guangdong Academy of Medical Sciences), Southern Medical University, Guangzhou, China

**Keywords:** Physical frailty, Aortic valve stenosis, Genetic predisposition, Prospective cohort, Longitudinal study

## Abstract

**Objectives:**

Cross-sectional evidence has implicated a high prevalence of frailty in patients with aortic valve stenosis (AS); however, the longitudinal association remains unknown. This study aimed to examine the longitudinal association between the physical frailty phenotype and the incidence of AS in middle-aged and older adults.

**Design:**

Prospective cohort and longitudinal study

**Setting:**

A population-based study of middle-aged and older adults.

**Participants:**

This study included participants from the UK biobank study.

**Measurements:**

Physical frailty was assessed using the Fried criteria frailty phenotype in the UK biobank in more than half a million participants. The primary outcome was incident degenerative AS, and the secondary outcome was AS-related events, that is, AS-related intervention or death due to AS. Cox proportional hazards models and competing risk models were used to evaluate their associations.

**Results:**

Among 480,967 participants (median age, 58.0 years; 54.6% female), 5,589 AS cases and 2,336 AS-related events were documented during a median follow-up of 14.3 years. Compared with robust participants, the adjusted hazard ratio (HR) in prefrail and frail participants was 1.30 (95% CI, 1.22−1.38) and 1.66 (95% CI, 1.50−1.84) for incident AS and 1.31 (95% CI, 1.19−1.43) and 1.54 (95% CI, 1.30−1.81) for AS-related events, respectively. The results were similar in a series of sensitivity analyses. Compared with robust participants with low genetic risk, frail participants with high genetic risk had the highest risk of AS (HR, 2.56; 95% CI, 2.15–3.06). Progression from robust to frail (HR, 2.41; 95% CI, 1.17−4.98) was associated with increased AS risk, while recovery from prefrail/frail to robust (HR, 0.35; 95% CI, 0.18−0.69) was associated with decreased AS risk.

**Conclusion:**

Physical prefrailty and frailty were associated with incident AS and subsequent AS-related events. These findings highlight the importance of integrating frailty assessment into the primary prevention of AS to better identify high-risk individuals.

## 1 Introduction

Degenerative aortic valve stenosis (AS) is the most common manifestation of valvular heart disease, with a global burden estimated at more than 9.4 million individuals [1]. This common cardiovascular condition predominantly affects individuals in their fifties and beyond, with its incidence rising due to an aging population and an increased prevalence of cardiovascular risk factors [1]. Nevertheless, there are no available medical therapies or recommended preventive measures to effectively treat AS, highlighting the urgent need to discern associated risk factors to facilitate early intervention [[1], [2], [3]]. However, few risk factors for AS have been recognized to date [2].

Frailty is a crucial clinical condition in both geriatric and cardiovascular spheres, as the prevalence and incidence of frailty and AS both markedly increase with age [4,5]. The physical frailty phenotype is characterized by increased vulnerability to stressors due to decreased physiologic reserve and decline of physical function across multiple physiological systems, which often leads to a variety of adverse health outcomes including multimorbidity, disability, and mortality [6]. Of these, the harmful effects of frailty on cardiovascular diseases have been well documented [[7], [8], [9]], but evidence of frailty in AS is very limited. It is plausible to anticipate that frailty may influence the development of AS, given that compelling evidence indicates a potential link between them, driven by common pathological mechanisms such as chronic inflammation, mechanical stress, oxidative stress, and metabolic dysregulation [5,[10], [11], [12]]. Previous research has highlighted that frailty has a high prevalence in patients with AS and always leads to poor clinical outcomes in patients undergoing valve replacement or repair [[13], [14], [15], [16]]. However, few studies have explored the longitudinal association between frailty and incident AS [17].

In addition, the occurrence and development of AS are influenced by genetic and acquired determinants [18,19], yet the modifying role of genetic predisposition in the association between frailty and AS risk remains largely unexplored [17]. While previous research has demonstrated that changes in frailty status are linked to the risk of incident cardiovascular diseases, it is still unclear whether such changes are associated with the risk of incident AS [17,20]. To fill these knowledge gaps, we sought to explore the longitudinal association between frailty and the risk of incident AS in the UK Biobank. We also examined whether genetic predisposition modified the association between frailty and AS. Finally, we investigated the associations of changes in frailty status with incident AS risk.

## 2 Materials and methods

### 2.1 Data sources

The UK Biobank is a large prospective cohort study that successfully recruited over half a million middle-aged and older adults from 22 centers across the UK between 2006 and 2010 [21]. It systematically gathers a diverse array of information, including sociodemographic characteristics, physical examinations, medical history, biological samples, and imaging data. Data were collected using various methods, such as digital and touch-screen questionnaires, oral interviews, clinical records, and physical measurements. This work has been reported in line with the STROCSS criteria [22].

### 2.2 Physical frailty assessment

We adopted the Fried’s phenotype to assess physical frailty, which is the most widely cited definition of frailty in epidemiological studies and is more clinically practical than the frailty index or frailty scale [5,23]. The frailty phenotype includes five criteria: weight loss, exhaustion, low physical activity, slow walking speed, and low grip strength. Several criteria definitions were modified to adapt to the availability of UK Biobank data, as previously reported [[23], [24], [25]]. Detailed definitions are provided in Supplementary materials, Table S1. For consistency with previous literature [[23], [24], [25]], participants were classified as robust (met none of the criteria), prefrail (met one or two criteria), or frail (met three or more criteria).

### 2.3 Assessment of outcomes

The primary outcome incident AS and the secondary outcome was AS-related events, that is, AS-related intervention or death due to AS. The ascertainment of AS and AS-related events was extracted from hospital episode records and death registers, determined using the International Statistical Classification of Diseases and Related Health Problems 10th Revision (ICD-10) codes and/or Office of Population Censuses and Surveys Classification of Interventions and Procedures 4th Revision (OPCS-4) codes, which were proven to have high accuracy in the diagnostic validity of AS, with a positive predictive value greater than 80% [26,27]. Diagnostic and procedural codes for degenerative AS and AS-related events are shown in Supplementary materials, Table S2 [27].

### 2.4 Polygenic risk score construction

The imputed genetic data were derived from the UK Biobank, and detailed information on genotyping and quality control has been described previously [28]. we comprehensively considered AS-related SNPs reported by Helgadottir et al. [29]. Three independent SNPs (r^2^ < 0.05 or 1000 kb apart, without linkage disequilibrium) were included [18], which were significantly associated with incident AS in individuals of European ancestry, with a minor allele frequency > 0.05 and *P* <  1 × 10^−5^. For each individual, we calculated polygenic risk score (PRS) as a weighted sum of the number of risk alleles based on the selected SNPs (information about the SNPs and PRS construction is provided in Supplementary materials, Methods). Participants with higher levels of PRS exhibited higher genetic susceptibility to AS, and were thus categorized into low (tertile 1), intermediate (tertile 2), and high (tertile 3) genetic risk groups.

### 2.5 Analytical cohorts

Three cohorts were formed based on distinct analytical purposes. The primary cohort (observational cohort) included 480,967 participants with valid frailty assessment data and no prevalent valvular heart disease, heart failure, or cardiomyopathy at baseline, as these conditions may reflect pre-existing or secondary valvular pathology and could obscure the identification of incident degenerative AS. Definitions for prevalent diseases are provided in Supplementary materials, Table S3. The baseline characteristics of participants stratified by the presence or absence of prevalent valvular heart disease are detailed in Supplementary Table S4, whereas those of included versus excluded participants are provided in Supplementary Table S5. The secondary cohort (genetic cohort) further excluded people without genetic data to construct the PRS for AS and comprised 391,432 participants. The third cohort (longitudinal cohort) was derived from the primary cohort and comprised participants with repeated frailty assessments at both baseline and the Imaging Visit (initiated on April 30, 2014) (N = 68,518). To ensure that frailty transitions preceded the outcome, individuals who developed AS prior to the Imaging Visit were excluded. Detailed inclusion and exclusion criteria are presented in Supplementary materials, Figure S1. For the primary and secondary cohorts, the follow-up periods were calculated from the enrollment date (baseline) to the occurrence of incident AS (primary outcome), AS-related events (secondary outcome), death, or the end of follow-up on June 30, 2023, whichever occurred first. In the longitudinal cohort, however, follow-up commenced at the date of the Imaging Visit, after the second frailty assessment was completed, to ensure proper temporality and avoid reverse causation in the analysis of frailty transition and subsequent AS risk.

### 2.6 Covariates

Based on prior literature [18,27], the study included a variety of covariates including age (years), sex (female or male), assessment center (England, Scotland, and Wales), race (White or non-White), Townsend deprivation index, education (college degree or not), smoking status (current smoker or not), healthy diet score (0−1, 2−3, and 4−5) (30), sleep duration (hours), systolic blood pressure (SBP, mmHg), glycated haemoglobin (HbA1c, mmol/mol), body mass index (BMI, kg/m^2^), C-reactive protein (CRP, mg/L), total cholesterol (TC, mmol/L), triglycerides (TG, mmol/L), low-density lipoprotein cholesterol (LDL-C, mmol/L), high-density lipoprotein cholesterol (HDL-C, mmol/L), estimated glomerular filtration rate (eGFR, mL/min/1.73 m^2^), prevalent comorbidities including atrial fibrillation, coronary heart disease, stroke, chronic obstructive pulmonary disease, osteoporosis, chronic inflammatory disease (psoriasis, systemic lupus erythematosus, and rheumatoid arthritis), antihypertensive medication, antidiabetic medication, lipid-lowering medication, and antiplatelet medication. The healthy diet score was constructed by assessing five dietary behaviors: daily consumption of at least four tablespoons of vegetables, intake of three or more pieces of fruit per day, fish consumption at least twice per week, and limiting both unprocessed and processed red meat to no more than twice per week. Participants received one point for each criterion met, yielding a total score ranging from 0 to 5, with higher scores reflecting healthier dietary patterns [30,31]. Detailed definitions on the prevalent comorbidities and medications are provided in Supplementary materials, Tables S3 and Table S6.

### 2.7 Statistical analysis

In the primary analysis, missing values for categorical covariates were coded as a separate category, while missing continuous covariates were imputed using mean values, a pragmatic approach commonly used in large-scale epidemiologic studies to ensure model stability and interpretability when covariates are used solely for adjustment [[32], [33], [34]]. Descriptive characteristics of the study participants were presented as mean (standard deviation [SD]), median (interquartile range [IQR]), or number (percentage), as appropriate. We reported the crude incidence rates of primary and secondary outcomes across the physical frailty groups, which are shown as the number of events per 100,000 person-years with time since frailty measurement as the timescale. We also plotted adjusted survival curves, based on Cox proportional hazard models and balanced for confounding variables in each subpopulation through marginal analysis, to depict the cumulative risk across different physical frailty groups (see Supplementary materials, Methods).

We assessed the proportional hazards assumption using Schoenfeld residuals and then utilized two Cox proportional hazards models to estimate the hazard ratio (HR) and corresponding 95% confidence interval (CI). Model 1 included sociodemographic factors and lifestyle, including age, sex, race, UK Biobank assessment center, Townsend deprivation index, education, smoking status, alcohol consumption status, healthy diet score, and sleep duration. Model 2 additionally incorporated BMI, SBP, HbA1c, TC, TG, LDL-C, HDL-C, eGFR, CRP, coronary heart disease, atrial fibrillation, stroke, chronic obstructive pulmonary disease, osteoporosis, chronic inflammatory disease, antihypertensive medication, antidiabetic medication, lipid-lowering medication, and antiplatelet medication.

Subgroup analyses were conducted in middle-aged (age < 60 years) and older (age ≥ 60 years) adults, and in male and female. We also examined the robustness of the results by conducting a series of sensitivity analyses: (i) participants who were diagnosed with incident AS or AS-related events within the initial 2 years of follow-up were excluded to combat potential reverse causality bias; (ii) participants with prevalent atrial fibrillation, coronary heart disease, or stroke at baseline were excluded to mitigate the influence of these cardiovascular comorbidities on the development of AS; (iii) the Fine and Gray competing risk model was fitted by treating death due to other causes as competing risks; and (iv) multiple imputation by chained equations was performed to impute missing covariate data to assess the robustness of the results and address limitations of single imputation methods (see Supplementary materials, Methods).

For the genetic analysis, we restricted the study population to the genetic cohort, and calculated *P* for interaction on a multiplicative scale by adding an interaction term in the model. We assessed the joint association of physical frailty and PRS with AS risk using Cox proportional hazards models, adjusting for covariates included in model 2. Finally, in the longitudinal cohort, we classified participants into the following categories to analyze the associations between changes in frailty status and incident AS: stable robust, robust to prefrail, robust to frail, stable prefrail/frail, and prefrail/frail to robust. All analyses were performed using R version 4.4.0. A two-sided *P* <  0.05 was considered statistically significant.

## 3 Results

### 3.1 Sample characteristics and incidence of outcomes

In the primary cohort of 480,967 individuals [median age (IQR): 58.0 (50.0–63.0) years; female: 54.6%], 227,328 (47.3) were robust, 229,162 (47.6%) were prefrail, and 24,477 (5.1%) were frail (Table 1). Participants with frailty tended to be older, female, had higher education, were more likely to be current smokers, and were less likely to have a healthy diet. They also had a higher body mass index, CRP, and HbA1c levels, and had a higher proportion of prevalent comorbidities and cardiovascular medications (Table 1). During a median follow-up of 14.3 years, there were 5,589 AS cases, along with 2,336 AS-related events. In the genetic cohort of 391,432 individuals [median age (IQR): 58.0 (50.0–63.0) years; female: 54.2%], 4,478 AS cases and 1,885 AS-related events occurred during a median follow-up of 14.3 years. In the longitudinal cohort of 68,518 individuals [median age (IQR): 55.0 (49.0–61.0) years; female: 51.7%], 221 AS cases and 76 AS-related events occurred during a median follow-up of 4.3 years.

**Table 1:** Baseline characteristics by physical frailty status in the UK Biobank.

	Total (*N* = 480,967)	Robust (*N* = 227,328)	Prefrail (*N* = 229,162)	Frail (*N* =24,477)	*P* value
Age (years)	58.0 (50.0–63.0)	57.0 (49.0–62.0)	59.0 (51.0–64.0)	59.0 (52.0–64.0)	< 0.001
Female, n (%)	262837 (54.6)	116762 (51.4)	130783 (57.1)	15292 (62.5)	< 0.001
Assessment center, n (%)					< 0.001
England	426565 (88.7)	199371 (87.7)	205509 (89.7)	21685 (88.6)	
Scotland	19963 (4.2)	9380 (4.1)	9440 (4.1)	1143 (4.7)	
Wales	34439 (7.2)	18577 (8.2)	14213 (6.2)	1649 (6.7)	
Race, White ethnicity, n (%)	453491 (94.3)	219389 (96.5)	212627 (92.8)	21475 (87.7)	< 0.001
Townsend deprivation index	−2.1 (-3.6 to 0.5)	−2.5 (-3.8 to -0.3)	−1.9 (-3.5 to 1.0)	0.0 (-2.6 to 3.2)	< 0.001
Education, college and above, n (%)	156052 (32.4)	86431 (38.0)	65557 (28.6)	4064 (16.6)	< 0.001
Current smoker, n (%)	50658 (10.5)	19989 (8.8)	25897 (11.3)	4772 (19.5)	< 0.001
Current drinker, n (%)	441738 (91.8)	215637 (94.9)	206774 (90.2)	19327 (79.0)	< 0.001
Healthy diet score, n (%)					< 0.001
0−1	63623 (13.2)	29686 (13.1)	30137 (13.2)	3800 (15.5)	
2−3	304923 (63.4)	145846 (64.2)	143732 (62.7)	15345 (62.7)	
4−5	107248 (22.3)	50704 (22.3)	51902 (22.6)	4642 (19.0)	
Missing	5173 (1.1)	1092 (0.5)	3391 (1.5)	690 (2.8)	
Sleep duration, hours	7.0 (7.0–8.0)	7.0 (7.0–8.0)	7.0 (6.0–8.0)	7.0 (6.0–8.0)	< 0.001
Systolic blood pressure, mmHg	137.8 (125.5–148.0)	137.8 (125.5–147.5)	137.8 (126.0–148.0)	137.8 (126.0–147.0)	< 0.001
Body mass index, kg/m^2^	26.8 (24.2–29.9)	25.9 (23.6–28.5)	27.5 (24.7–30.7)	30.1 (26.4–34.5)	< 0.001
C-reactive protein, mg/L	1.4 (0.7–2.6)	1.2 (0.6–2.5)	1.7 (0.8–3.0)	2.6 (1.3–5.2)	< 0.001
TC, mmol/L	5.7 (5.0–6.4)	5.7 (5.1–6.4)	5.7 (4.9–6.3)	5.5 (4.6–6.2)	< 0.001
TG, mmol/L	1.6 (1.1–2.1)	1.5 (1.0–2.0)	1.6 (1.1–2.1)	1.7 (1.3–2.4)	< 0.001
HDL-C, mmol/L	1.4 (1.2–1.6)	1.4 (1.2–1.7)	1.4 (1.2–1.6)	1.4 (1.1–1.5)	< 0.001
LDL-C, mmol/L	3.6 (3.0–4.1)	3.6 (3.0–4.1)	3.6 (3.0–4.1)	3.4 (2.8–3.9)	< 0.001
Glycated haemoglobin, mmol/mol	35.5 (33.0–37.6)	35.1 (32.6–37.1)	36.0 (33.3–38.1)	36.4 (34.3–40.4)	< 0.001
Estimated GFR, mL/min/1.73 m^2^	91.8 (83.6–99.4)	92.0 (83.9–99.5)	91.6 (83.5–99.2)	91.3 (81.8–99.7)	< 0.001
Atrial fibrillation, n (%)	6507 (1.4)	2543 (1.1)	3345 (1.5)	619 (2.5)	< 0.001
Coronary heart disease, n (%)	16915 (3.5)	5352 (2.4)	9248 (4.0)	2315 (9.5)	< 0.001
Stroke, n (%)	7443 (1.5)	1961 (0.9)	4189 (1.8)	1293 (5.3)	< 0.001
Chronic obstructive pulmonary disease, n (%)	9062 (1.9)	2244 (1.0)	4942 (2.2)	1876 (7.7)	< 0.001
Osteoporosis, n (%)	10130 (2.1)	3572 (1.6)	5410 (2.4)	1148 (4.7)	< 0.001
Chronic inflammatory disease, n (%)	17390 (3.6)	5798 (2.6)	9314 (4.1)	2278 (9.3)	< 0.001
Psoriasis, n (%)	10630 (2.2)	4418 (1.9)	5376 (2.3)	836 (3.4)	< 0.001
Systemic lupus erythematosus, n (%)	727 (0.2)	213 (0.1)	369 (0.2)	145 (0.6)	< 0.001
Rheumatoid arthritis, n (%)	6467 (1.3)	1221 (0.5)	3815 (1.7)	1431 (5.8)	< 0.001
Antihypertensive medication, n (%)	107491 (22.3)	37558 (16.5)	59391 (25.9)	10542 (43.1)	< 0.001
Antidiabetic medication, n (%)	18093 (3.8)	3921 (1.7)	10960 (4.8)	3212 (13.1)	< 0.001
Lipid-lowering medication, n (%)	85586 (17.8)	29729 (13.1)	47128 (20.6)	8729 (35.7)	< 0.001
Antiplatelet medication, n (%)	65233 (13.6)	24707 (10.9)	34527 (15.1)	5999 (24.5)	< 0.001

Continuous variables are presented as median (interquartile range) owing to non-normal distribution, and categorical variables are presented as n (%).

TC, total cholesterol; TG, triglycerides; LDL-C, low-density lipoprotein cholesterol; HDL-C, high-density lipoprotein cholesterol; GFR, glomerular filtration rate.

### 3.2 Association of physical frailty with incident AS and AS-related events

**Fig. 1:**
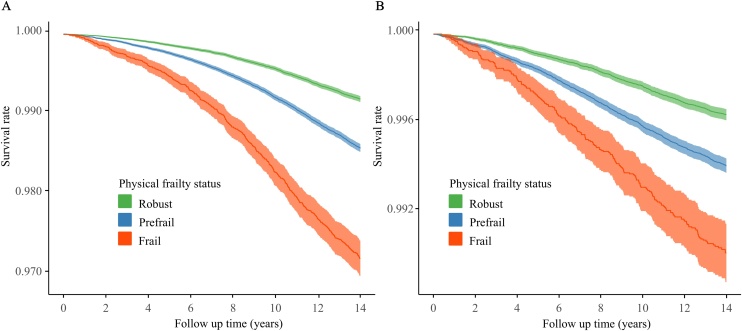
Adjusted survival curves for the risk of (A) aortic valve stenosis and (B) aortic valve stenosis-related events across groups of physical frailty status. Adjusted survival curves were estimated using Cox proportional hazard models, in which adjustments were made for age, sex, race, UK Biobank assessment center, Townsend deprivation index, education, smoking status, alcohol consumption status, healthy diet score, sleep duration, body mass index, systolic blood pressure, glycated haemoglobin, total cholesterol, triglycerides, low-density lipoprotein cholesterol, high-density lipoprotein cholesterol, estimated glomerular filtration rate, C-reactive protein, coronary heart disease, atrial fibrillation, stroke, chronic obstructive pulmonary disease, osteoporosis, chronic inflammatory disease, antihypertensive medication, antidiabetic medication, lipid-lowering medication, and antiplatelet medication.

The adjusted survival curves for AS and AS-related events across the physical frailty groups are displayed in Fig. 1. The incidence rates of AS were 5.70, 9.95, and 19.52 per 10,000 person-years in robust, prefrail, and frail participants, respectively, with the corresponding values for AS-related events being 2.54, 4.14, and 6.77, respectively (Table 2). Compared with the robust group, the fully-adjusted HRs for AS and AS-related events were 1.30 (95% CI: 1.22−1.38) and 1.31 (95% CI: 1.19−1.43) for the prefrail group, and 1.66 (95% CI: 1.50−1.84) and 1.54 (95% CI: 1.30−1.81) for the frail group, respectively (*P* for trend < 0.001) (Table 2). We also found that individual components of frailty were associated with incident AS and AS-related events. In the fully adjusted model, weight loss (HR, 1.30; 95% CI, 1.21−1.39), exhaustion (HR, 1.16; 95% CI, 1.07−1.25), slow gait speed (HR, 1.30; 95% CI, 1.21−1.41), and low grip strength (HR, 1.18; 95% CI, 1.11−1.25) were significantly associated with incident AS, while a null association was observed for low physical activity. The corresponding HRs with 95% CI for AS-related events for weight loss, exhaustion, low physical activity, slow gait speed, and low grip strength were 1.22 (1.10−1.36), 1.10 (0.97−1.24), 1.10 (0.97−1.24), 1.16 (1.02−1.31), and 1.23 (1.12−1.34), respectively (Table 3).

**Table 2:** Association of physical frailty status with risk of incident degenerative AS and AS-related events.

	Robust	Prefrail	Frail	*P* for trend *
AS				
Case/N	1824/227,328	3142/229,162	623/24,477	
Incident rate per 100,000 person-years	5.70	9.95	19.52	
Model 1, HR (95% CI)^a^	Reference	1.55 (1.47−1.65)	2.91 (2.65−3.20)	< 0.001
Model 2, HR (95% CI)^b^	Reference	1.30 (1.22−1.38)	1.66 (1.50−1.84)	< 0.001
AS-related events				
Case/N	812/227,328	1308/229,162	216/24,477	
Incident cases per 100000 person-years	2.54	4.14	6.77	
Model 1, HR (95% CI)^a^	Reference	1.52 (1.39−1.66)	2.47 (2.11−2.88)	< 0.001
Model 2, HR (95% CI)^b^	Reference	1.31 (1.19−1.43)	1.54 (1.30−1.81)	< 0.001

HR, hazard ratio; CI, confidence interval; AS, aortic valve stenosis.

a Model 1 adjusted for age, sex, race, UK Biobank assessment center, Townsend deprivation index, education, smoking status, alcohol consumption status, healthy diet score, and sleep duration.

b Model 2 included model 1 plus body mass index, systolic blood pressure, glycated haemoglobin, total cholesterol, triglycerides, low-density lipoprotein cholesterol, high-density lipoprotein cholesterol, estimated glomerular filtration rate, C-reactive protein, coronary heart disease, atrial fibrillation, stroke, chronic obstructive pulmonary disease, osteoporosis, chronic inflammatory disease, antihypertensive medication, antidiabetic medication, lipid-lowering medication, and antiplatelet medication.

* *P* for trend was calculated using the median physical frailty score (0, 1 and 3) for each frailty status.

**Table 3:** Association of individual components of physical frailty with risk of incident degenerative AS and AS-related events.

Physical frailty components	HR (95% CI)		
	Model 1 a	Model 2 b	Model 3 c
AS			
Weight loss	1.38 (1.29−1.47)	1.29 (1.21−1.38)	1.30 (1.21−1.39)
Exhaustion	1.54 (1.43−1.66)	1.22 (1.13−1.32)	1.16 (1.07−1.25)
Low physical activity	1.46 (1.35−1.58)	1.12 (1.04−1.21)	1.04 (0.96−1.12)
Slow gait speed	2.19 (2.04−2.35)	1.37 (1.27−1.47)	1.30 (1.21−1.41)
Low grip strength	1.46 (1.39−1.55)	1.20 (1.14−1.27)	1.18 (1.11−1.25)
AS-related events			
Weight loss	1.28 (1.15−1.43)	1.22 (1.09−1.35)	1.22 (1.10−1.36)
Exhaustion	1.39 (1.23−1.57)	1.14 (1.01−1.29)	1.10 (0.97−1.24)
Low physical activity	1.44 (1.28−1.62)	1.15 (1.02−1.30)	1.10 (0.97−1.24)
Slow gait speed	1.85 (1.65−2.07)	1.23 (1.09−1.39)	1.16 (1.02−1.31)
Low grip strength	1.46 (1.34−1.58)	1.24 (1.14−1.35)	1.23 (1.12−1.34)

HR, hazard ratio; CI, confidence interval; AS, aortic valve stenosis.

a Model 1 adjusted for age, sex, race, UK Biobank assessment center, Townsend deprivation index, education, smoking status, alcohol consumption status, healthy diet score, and sleep duration.

b Model 2 included model 1 plus body mass index, systolic blood pressure, glycated haemoglobin, total cholesterol, triglycerides, low-density lipoprotein cholesterol, high-density lipoprotein cholesterol, estimated glomerular filtration rate, C-reactive protein, coronary heart disease, atrial fibrillation, stroke, chronic obstructive pulmonary disease, osteoporosis, chronic inflammatory disease, antihypertensive medication, antidiabetic medication, lipid-lowering medication, and antiplatelet medication.

c Model 3 included model 2 plus five frailty components (mutual adjustment).

### 3.3 Subgroup and sensitivity analysis

In the stratified analyses, the association of physical frailty and individual components of frailty with the risk of incident AS and AS-related events remained mostly consistent when stratified by age (< 60 years and ≥ 60 years) and sex (male and female) (Supplementary materials, Tables S7-S8). In addition, we observed effect modifications of age on the associations between physical frailty and the risk of incident AS (*P* for interaction < 0.05), and modifications of sex on the associations between physical frailty and the risk of incident AS-related events (*P* for interaction < 0.05), where the associations were stronger in middle-aged than in older adults, and in women than in men (Tables S7). Similarly, the associations of exhaustion with the risk of AS were also stronger in middle-aged than older adults, while the associations of slow gait speed with risk of AS were stronger in women than in men (Supplementary materials, Tables S8). The main results did not change significantly across various sensitivity analyses. Excluding participants with incident outcomes within 2 years of follow-up (Supplementary materials, Tables S9), excluding participants with prevalent atrial fibrillation, coronary heart disease, or stroke at baseline (Supplementary materials, Tables S10), accounting for competing risks from other causes of death (Supplementary materials, Tables S11), and imputing missing covariate using multiple imputation (Supplementary materials, Tables S12) resulted in slightly changed associations.

### 3.4 Genetic analysis

**Fig. 2:**
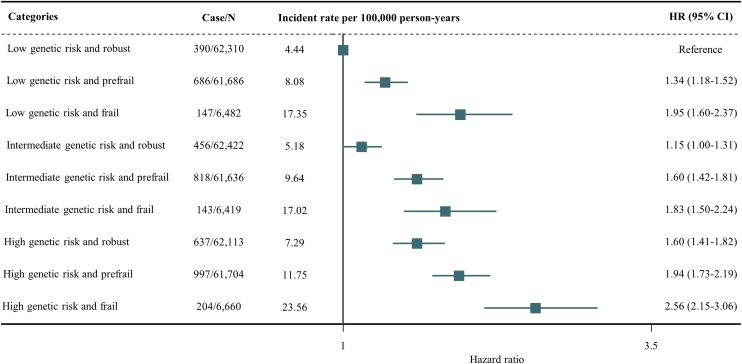
Joint associations of physical frailty status and polygenetic risk score with the risk of incident aortic valve stenosis. HR, hazard ratio; CI, confidence interval. The risks were estimated using Cox proportional hazard models adjusted for age, sex, race, UK Biobank assessment center, Townsend deprivation index, education, smoking status, alcohol consumption status, healthy diet score, sleep duration, body mass index, systolic blood pressure, glycated haemoglobin, total cholesterol, triglycerides, low-density lipoprotein cholesterol, high-density lipoprotein cholesterol, estimated glomerular filtration rate, C-reactive protein, coronary heart disease, atrial fibrillation, stroke, chronic obstructive pulmonary disease, osteoporosis, chronic inflammatory disease, antihypertensive medication, antidiabetic medication, lipid-lowering medication, and antiplatelet medication.

Participants categorized as having intermediate or high genetic risk had an increased risk of incident AS, with HRs of 1.15 (95% CI, 1.07–1.24) and 1.49 (1.38–1.60), respectively, compared with those with low genetic risk (Supplementary materials, Table S13). Using the robust group as reference, participants in the prefrail and frail group had increased risk of incident AS across low-, intermediate-, and high-PRS groups (Supplementary materials, Table S14). We observed a multiplicative interaction between physical frailty and genetic predisposition for AS risk (*P* for interaction = 0.033). The joint association of physical frailty and PRS with AS risk is presented in Fig. 2, where a cumulative association was found. The findings indicated that, compared with robust participants with low exposure genetic risk, the most substantial risk of AS was found in frail participants exposed to high genetic risk (HR, 2.56; 95% CI, 2.15–3.06).

### 3.5 Association of change in physical frailty status with incident AS

Table 4 shows the association between changes in frailty status and risk of incident AS. Compared to participants who remained robust, those who progressed to frailty showed significantly increased AS risk (HR 2.41, 95% CI 1.17−4.98). In contrast, significantly decreased risks were observed in the prefrail/frail participants who recovered to a robust status when compared with stable prefrail/frail participants (HR 0.35, 95% CI 0.18−0.69).

**Table 4:** Association of changes in physical frailty status with risks of incident AS (N = 68,518).*

		Robust at baseline (N = 39,671)		Prefrail/frail at baseline (N = 28,847)	
	Stable robust	Robust to prefrail	Robust to frail	Stable prefrail/frail	Prefrail/frail to robust
	Reference	0.90 (0.62−1.33)	2.41 (1.17−4.98)	Reference	0.35 (0.18−0.69)

HR, hazard ratio; CI, confidence interval; AS, aortic valve stenosis.

* Models were adjusted for age, sex, race, UK Biobank assessment center, Townsend deprivation index, education, smoking status, alcohol consumption status, healthy diet score, sleep duration, body mass index, systolic blood pressure, glycated haemoglobin, total cholesterol, triglycerides, low-density lipoprotein cholesterol, high-density lipoprotein cholesterol, estimated glomerular filtration rate, C-reactive protein, coronary heart disease, atrial fibrillation, stroke, chronic obstructive pulmonary disease, osteoporosis, chronic inflammatory disease, antihypertensive medication, antidiabetic medication, lipid-lowering medication, and antiplatelet medication.

## 4 Discussion

We examined the association of the physical frailty phenotype with incident degenerative AS and related events among middle-aged and older adults in the UK biobank during a 14-year follow-up. In this study, we offered novel insights into the heightened risk of degenerative AS associated with physical frailty and found that prefrail and frail individuals had a 1.30-fold and 1.66-fold increased risk of incident AS, as well as a 1.31-fold and 1.54-fold increased risk of incident AS-related events, respectively. Additionally, we observed an interaction between frailty and genetic susceptibility on the incidence of AS, with the highest risk observed in those with both frailty and high PRS. Finally, we confirmed that different changes in frailty status were associated with varying risks of incident AS.

Previous studies have found that frailty is common in adults with AS. In the Frailty Assessment Before Cardiac Surgery & Transcatheter Interventions (FRAILTY-AVR) study of 1,020 older patients undergoing transcatheter or surgical aortic valve replacement, the prevalence of frailty was 26%–68% based on multiple frailty scales, of which 40% were frail according to the Fried phenotype [16]. Similarly, a study using the Fried criteria to assess frailty showed that the prevalence rate was 45.5% among patients undergoing percutaneous aortic valve repair [35]. These findings support those of other studies using alternative indices to measure frailty in patients with AS, showing the high prevalence of frailty. Shimura et al. [36] applied the Clinical Frailty Scale to evaluate frailty in 1,215 patients who underwent transcatheter aortic valve replacement and found that 29.1% of participants were classified as frail. Another study conducted by Kundi et al. [15] found that the intermediate or high risk for frailty defined by the Hospital Frailty Risk Score, an ICD-10 claims-based score, was present in 49.2% and 47.5% of the participants who underwent transcatheter mitral valve repair and transcatheter aortic valve replacement, respectively. In a cohort of older patients with severe aortic stenosis, V. Arnold et al. [14] examined the prevalence of frailty as a continuous variable based on four markers including grip strength, gait speed, serum albumin, and activities of daily living, and reported that 67.5% and 6.1% were prefrail (1–2 frailty markers) and 6.1% were frail (3–4 frailty markers). However, little longitudinal evidence has been reported regarding physical frailty and incident AS. In our research, prefrailty and frailty were not only significantly associated with an increased risk of incident AS, but also had a close relationship with AS-related interventions or death due to AS. These associations were robust after adjusting for sociodemographic factors, lifestyles, multiple morbidities, and genetic background.

Our study also found that the components of frailty, including weight loss, exhaustion, slow gait speed, and low grip strength, but not low physical activity, may correlate with AS and AS-related events. Among the 5 components, slow gait speed demonstrated the most significant association with AS risk. Multiple studies have linked frailty components with the risk of cardiovascular diseases, such as coronary heart disease and stroke [5,7,8]. Our study adds essential evidence for the impact of frailty components on risk of AS. In addition, the prominent association of frailty, as well as components of frailty, and AS risk was observed among middle-aged and female individuals, suggesting that younger, female individuals may be more susceptible to frailty for incident AS. We also utilized PRS to explore the link between PRS for AS and the frailty phenotype in AS incidence. Our findings revealed that a greater risk of developing AS was linked to poorer frailty status and elevated AS-PRS. Moreover, a significant interaction between frailty and PRS categories was identified in the joint analyses, indicating a gene-environment interaction in the occurrence of AS. These results imply that enhancing physical frailty could benefit individuals, even those with high genetic predisposition to AS. Consequently, public health strategies aimed at improving physical capabilities could help lower the burden of frailty and reduce the risk of AS associated with frailty.

The exact mechanism by which frailty contributes to the development of degenerative AS remains poorly understood. It is possible that frailty is often linked to reduced physical activity and poor dietary habits, further increasing the risk associated with degenerative AS. This lifestyle is correlated with factors such as metabolic imbalance, enhanced platelet activation, and arteriosclerosis, all of which are associated with degenerative AS [[37], [38], [39]]. Additionally, frailty initiates inflammatory processes and coincides with chronic systemic inflammation, which can lead to mitochondrial dysfunction and increased oxidative stress, cause pathological alterations in heart valves, negatively affect heart valve function, and encourage valve calcification [[40], [41], [42]]. Studies in large populations have also indicated that frail elderly individuals face higher risks of various cardiovascular conditions, including hypertension and high lipid levels, and the accumulation of these factors heightens the susceptibility of the cardiovascular system to degenerative AS [43,44]. Despite these insights, more research is needed to fully elucidate the mechanisms connecting different frailty categories to AS, which could inform the creation of specific interventions aimed at reducing AS risk in prefrail and frail populations.

Our research carries significant implications for the prevention of AS. Currently, the prevalence of degenerative AS is rapidly increasing worldwide; however, the lack of pharmacological therapies and treatment methods has historically limited to the surgical techniques of valve repair or replacement [45]. A substantial body of evidence suggests that frailty is associated with adverse outcomes in patients with AS [5,[13], [14], [15], [16],^35^,^36^], which causes that a considerable proportion of frail patients to be ineligible for surgical interventions. Current guidelines, such as the European Society of Cardiology (ESC)/European Association for Cardio-Thoracic Surgery (EACTS) and the American College of Cardiology (ACC)/American Heart Association (AHA) guidelines for the management of valvular heart disease, mainly advocate frailty assessment as a tertiary prevention measures, and treat frailty as an important candidate for optimizing patient selection for cardiac interventional procedures [46,47]. Our study provides novel insights into the crucial role of frailty screening in the primary prevention of AS, by claiming that physical prefrailty and frailty are both closely associated with incident AS. In addition, our study found consistent associations between frailty and AS across age and sex subgroups, even across different genetic risks, and confirmed that progression from robustness to frailty was related to increased AS risk, while transition from prefrail or frail status to robust was associated with in a decreased risk. These results suggest that prefrail and frail individuals should be the primary focus for preventing AS, while robust individuals should also assess frailty risk factors to identify high-risk individuals. Taken together, our findings suggest that frailty assessment should be integrated into routine cardiovascular practice and considered a potential non-pharmaceutical intervention to halt or slow the progression of degenerative AS.

Our study has several strengths. First, drawing upon a nationwide cohort, we provide prospective evidence of the longitudinal association between physical frailty and incident AS as well as AS-related events. The large sample size (over 500,000 individuals) and long-term follow-up period (up to 14 years) increased the credibility of our results. Second, we innovatively explored the impact of frailty state transitions on AS risk, which is essential for the development of future interventional measures.

However, we also acknowledge that this study is subjected to several potential limitations. First, asymptomatic cases might have been underdiagnosed because our work relied solely on ICD-10 diagnosis codes to capture AS cases, which were primarily at advanced stages. This could have led us to underestimate the disease risk related to physical frailty; additionally, we were unable to assess disease progression or severity. Moreover, the absence of echocardiographic data for direct case ascertainment could cause some degree of misclassification of disease outcomes. However, previous studies have proven the high diagnostic validity of electronic health records compared with echocardiographic assessment for recognizing moderate to severe cases [43,44,48]. In addition, we set AS-related interventions or death due to AS as secondary outcomes representing disease severity and yielded similar results. Second, the frailty phenotype was adapted from the original Fried criteria using measures available in the UK Biobank, with four of the five components derived from self-reported data. This may introduce recall or reporting bias, particularly for subjective indicators such as weight loss and exhaustion. Nonetheless, this approach has been consistently applied across large-scale UK Biobank studies [[23], [24], [25],^49^], and prior research has shown that self-reported frailty indicators yield comparable prevalence estimates and associations with health outcomes to those based on objective assessments [50]. These findings support the construct validity of the adapted definition. Moreover, the robustness of our results and the inclusion of objectively measured grip strength further strengthen its reproducibility in this setting. Third, owing to the observational nature of the study design, our study had inherent limitations for establishing causal associations and insufficient ability to dispose of residual confounding from unknown or unmeasured factors, although a broad range of confounders have been incorporated into consideration. Fourth, the UK Biobank might be subject to selection bias due to its low response rate (5.47%) [51], even though the effect sizes estimated from the UK Biobank appear to be comparable to those from population-representative cohorts, according to previous research [52]. Fifth, the study was limited to individuals of European ancestry. Thus, further research is warranted to replicate our results in other ethnicities.

## 5 Conclusion

In summary, our results suggest that physical frailty is associated with an increased risk of AS and subsequent adverse events, and that reversal of frailty may be effective in preventing them. Our findings highlight the importance of comprehensively assessing physical frailty for the primary prevention of AS. Future research is needed to develop precise prevention strategies to delay frailty progression as well as tailored interventions to reverse frailty in cardiovascular practice.

## CRediT authorship contribution statement

BBS, CLC and TMS conceived the study. BBS wrote the first draft. BBS, PPZ, ZHS, and TMS interpreted the data and critically revised the manuscript for intellectual content. All authors read and approved the final manuscript. TMS supervised the study and had the final responsibility in deciding to submit for publication.

## Ethical standards

Ethical approval was provided by the North West Multicenter Research Ethics Committee (reference number: 11/NW/0382), with all participants signing written informed consent.

## Declaration of Generative AI and AI-assisted technologies in the writing process

No AI was used in the writing process.

## Funding

This work was supported by the National Natural Science Foundation of China (NSFC) Young Scientists Fund (82300339), and Guangdong Provincial Medical Science and Technology Research Fund (A2024141).

## Declaration of competing interest

The authors declared no potential conflicts of interest.
