# Supplementary material for: Physical frailty, genetic predisposition, and risk of incident degenerative aortic valve stenosis: A prospective cohort study
Source: J Nutr Health Aging. 2025 Aug 6;29(10):100648. doi: 10.1016/j.jnha.2025.100648 (PMC12355512; doi:10.1016/j.jnha.2025.100648)
Supplement: Supplementary file 1 [file mmc1.docx]

**Supplementary materials**

**Supplementary Methods**

**Construction of the** **polygenic risk score for AS**

The polygenic risk score (PRS) for AS was calculated as follows:

$$Weighted PRS= \sum_{i=1}^{n} \beta_{i}{SNP}_{i}\times(\frac{N}{Sum(\beta)})$$

Where $\beta$ is per allele log odds ratios for AS associated with $SNP$, which obtained from the previous GWASs; $SNP$ was documented as 0, 1, and 2 according to the number of risk alleles; $n$ was the total of selected $SNPs$. The three independent SNPs used for PRS construction are summarized as follows:

| Chromosome | rsID | Position | Effect allele | Odds ratios |
| --- | --- | --- | --- | --- |
| 1 | rs7543130 | 5747184 | A | 1.20 |
| 2 | rs1830321 | 145825555 | T | 1.15 |
| 6 | rs10455872 | 161010118 | G | 1.46 |

**Adjusted survival curves**

We utilized the “ggadjustedcurves” function in R to generate adjusted survival curves based on Cox proportional hazards models. In Cox proportional hazards models, we used follow-up time since baseline as the time variable to define the risk sets for outcomes and adjustments were made for covariates, including sex, ethnicity, Townsend deprivation index, education, smoking status, alcohol intake, diet score, sleep duration, and discretionary screen-time. The formula is expressed as follows:

$$h\left( a | z_{i} \right)=h_{0}(a)exp(\beta z_{i})$$

where $\boldsymbol{a}$ is the age at which participants exited the analysis, either at the time of the occurrence of AS (primary outcome), AS-related intervention or death (secondary outcome), death from other causes, or the end of follow-up on June 30, 2023, whichever came first; $\boldsymbol{z}_{\boldsymbol{i}}$represents the levels of other covariates; $\boldsymbol{\beta}$ is the regression coefficient of the other covariates. By applying the marginal analysis method, we derived adjusted survival curves that offer a balanced representation for each group concerning the specified covariates. The balancing process is as follows: (1) logistic regression models are constructed for each subpopulation to predict the odds of belonging to that group compared to the whole population, taking into account other variables, (2) reverse probabilities of belonging to a specified subpopulation are used as weights in the Cox models, (3) the Cox models are refitted with weights taken into account, (4) expected survival curves are then computed for each subpopulation based on the refitted Cox models with applied weights.

**Multiple imputation for missing data**

Missing covariate data were addressed using multiple imputation by chained equations in R software (version 4.3.3), assuming that the missing data were at random. The covariates included in the imputation models were variables used in the analysis models plus candidate predictors, and outcome variables. To reduce random error, we imputed 30 datasets and then combined estimates using Rubin's rules. The covariates included in the imputation models were age (continuous), sex (dichotomous), race (dichotomous), UK Biobank assessment center (categorical),Townsend deprivation index(continuous), education(dichotomous), smoking status (dichotomous), alcohol consumption status (dichotomous), healthy diet score (ordinal), sleep duration (continuous), body mass index (continuous), systolic blood pressure (continuous), glycated haemoglobin (continuous), total cholesterol (continuous), triglycerides (continuous), low-density lipoprotein cholesterol (continuous), high-density lipoprotein cholesterol (continuous), estimated glomerular filtration rate (continuous), C-reactive protein (continuous), coronary heart disease (dichotomous), atrial fibrillation (dichotomous), stroke (dichotomous), chronic obstructive pulmonary disease (dichotomous), osteoporosis (dichotomous), chronic inflammatory disease (dichotomous), antihypertensive medication (dichotomous), antidiabetic medication (dichotomous), Lipid-lowering medication (dichotomous), aspirin use (dichotomous), death event (dichotomous), aortic valve stenosis (dichotomous), AS-related events (dichotomous), and event date (Continuous; cumulative hazard rate transformed).

**Figure S1 Flowchart of the study.**

**
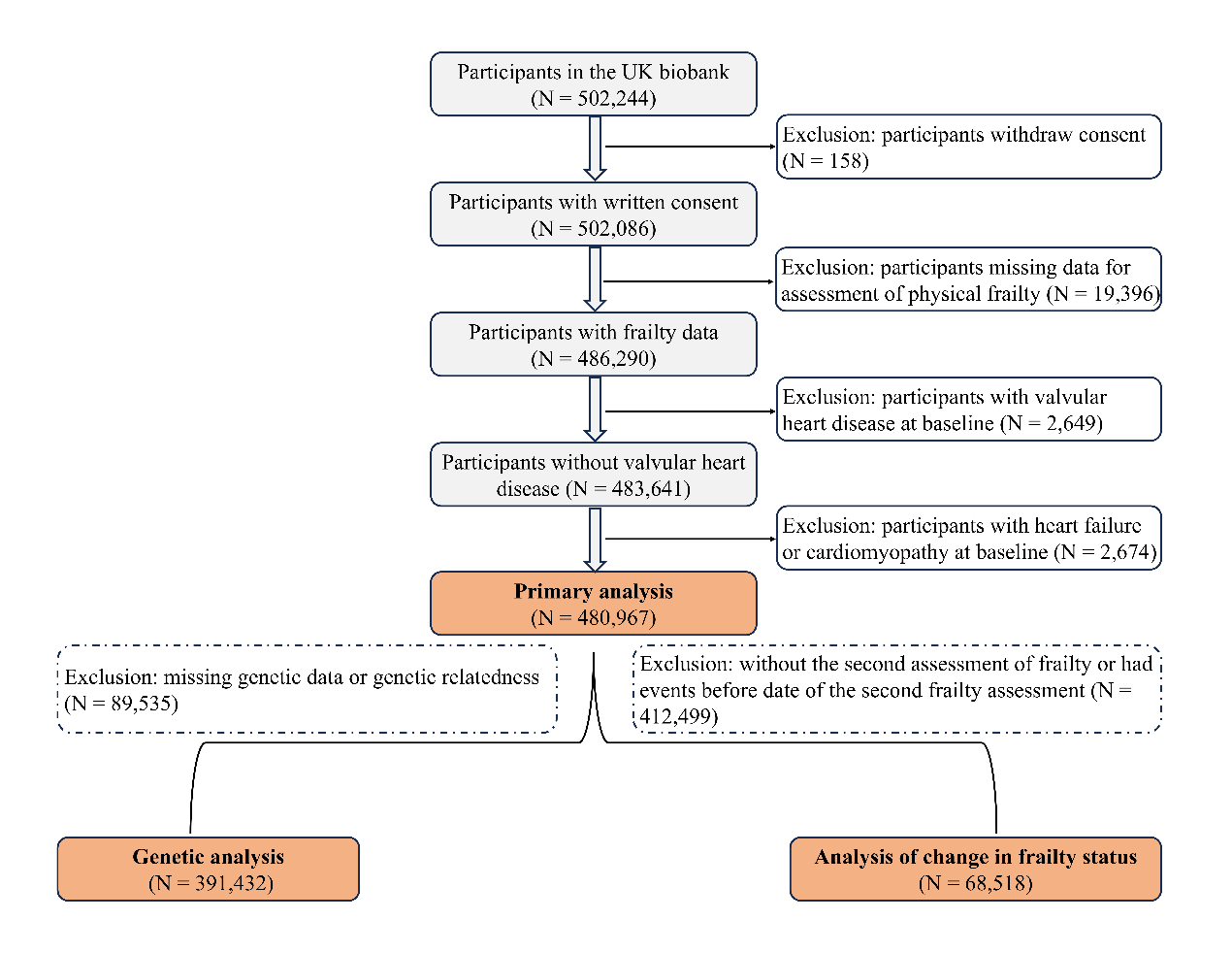
**

AS, aortic valve stenosis.

**Table S1. The list of physical frailty definition in the UK Biobank**

| **Components** | **Definition** | **Field ID** |
| --- | --- | --- |
| Weight loss | Self-reported: “Compared with one year ago, has your weight changed?” Response: yes, lost weight = 1; other = 0; Do not know/Prefer not to answer = missing data. | 2306 |
| Exhaustion | Self-reported: “Over the past two weeks, how often have you felt tired or had little energy?” Response: more than half the days or nearly every day = 1; other = 0; Do not know/Prefer not to answer = missing data. | 2080 |
| Low physical activity | Self-reported: "In the last 4 weeks did you spend any time doing the following" Response:  Light DIY (eg: pruning, watering the lawn): frequency of once per week or less = 1, more than once a week = 0 None of above = 1 Walking for pleasure (not as a means of transport) = 0 Other exercises (eg: swimming, cycling, keep fit, bowling) = 0 Strenuous sports = 0 Heavy DIY (eg: weeding, lawn mowing, carpentry, digging) = 0 Prefer not to answer (Excluded) | 6164, 1011 |
| Slow walking speed | Self-reported: “How would you describe your usual walking pace?” Response: slow = 1; other = 0; Do not know/Prefer not to answer = missing data. | 924 |
| Low grip strength | Measured grip strength expressed in kg by sex- and BMI- adjusted cut-off points.  Cut-off points:  Men  If BMI ≤ 24.0 kg/m^2^ & grip strength ≤ 29 kg  If BMI 24.1 to 26.0 kg/m^2^ & grip strength ≤ 30 kg  If BMI 26.1 to 28.0 kg/m^2^ & grip strength ≤ 30 kg  If BMI > 28.0 kg/m^2^ & grip strength ≤ 32 kg  Women If BMI ≤ 23.0 kg/m^2^ & grip strength ≤ 17 kg  If BMI 23.1 to 26.0 kg/m^2^ & grip strength ≤ 17.3 kg If BMI 26.1 to 29.0 kg/m^2^ & grip strength ≤ 18 kg  If BMI > 29.0 kg/m^2^ & grip strength ≤ 21 kg If data on BMI or grip strength is not available = missing data. | 31, 21001, 46, 47 |

BMI, body mass index.

**Table S2. ICD-10 codes and Field ID defining outcomes in UK biobank**

| **Outcome** | **Codes** | **Data field ID** |
| --- | --- | --- |
| AS | I35.0; I35.2 (ICD-10 codes from hospital episode records or death registers) | 41270; 40001 |
| AS-related intervention or death due to AS | [I35.0 (hospital episode records) & K26 (hospital operation records)] or  [I35.2 (hospital episode records) & K26 (hospital operation records)] or  I35.0 (death registers) or I35.2 (death registers) | 41270; 41272; 40001  P40001; P40002; 40001 |

AS, aortic valve stenosis; ICD: international classification disease; OPCS: Office of Population Censuses and Surveys Classification of Interventions and Procedures.

(K26 Plastic repair of aortic valve-K26.1 Allograft replacement of aortic valve; K26.2 Xenograft replacement of aortic valve; K26.3 Prosthetic replacement of aortic valve; K26.4 Replacement of aortic valve NEC; K26.5 Aortic valve repair NEC; K26.8 Other specified plastic repair of aortic valve;K26.9 Unspecified plastic repair of aortic valve).

**Table S3. ICD-10 codes and Field ID defining** **prevalent diseases at baseline in UK biobank**

| **Diseases** | **ICD-10 codes** | **Data field ID** |
| --- | --- | --- |
| **Valvular heart disease at baseline** |  |  |
| **Rheumatic valve disease** |  |  |
| Mitral valve | I05.0; I05.1; I05.2; I05.8; I05.9; | 131276 |
| Aortic valve | I06.0; I06.1; I06.2; I06.8; I06.9; | 131278 |
| Tricuspid valve | I07.0; I07.1; I07.2; I07.8; I07.9; | 131280 |
| **Congenital valve disease or Marfan's syndrome** |  |  |
| Pulmonary and tricuspid valves | 132470 | Q22.1; Q22.2; Q22.4; Q22.5; Q22.8; Q22.9; |
| Aortic and mitral valves | 132472 | Q23.0; Q23.1; Q23.2; Q23.3; Q23.4; Q23.8; Q23.9; |
| Marfan's syndrome | 41270 | Q87.4 |
| **Endocarditis with Valvular Heart Disease** |  |  |
| Valve unspecified | 131330 | I38; |
| Heart valve disorders in diseases classified elsewhere | 131332 | I39.0; I39.1; I39.3; I39.4; I39.8; |
| **Nonrheumatic heart diseases** |  |  |
| Mitral valve disorders | 131322 | I34.0; I34.1; I34.2; I34.8; I34.9; |
| Aortic valve disorders | 131324 | I35.0; I35.1; I35.2; I35.8; I35.9; |
| Tricuspid valve disorders | 131326 | I36.0; I36.1; I36.2; I36.8; I36.9; |
| Pulmonary valve disorders | 131328 | I37.0; I37.1; I37.2; I37.8; I37.9; |
| **Multiple valve disorders** | 131382 | I08.0; I08.1; I08.2; I08.3; I08.8; I08.9; |
| **Comorbidities at baseline** |  |  |
| **Heart failure** | I50.0; I50.1; I50.9; | 131354 |
| **Cardiomyopathy** | I42.0; I42.1; I42.2; I42.3; I42.4; I42.5; I42.6; I42.7; I42.8; I42.9; | 131338 |
| **Atrial fibrillation** | I48.0; I48.1; I48.2; I48.3; I48.4; I48.9 | 131350 |
| **Coronary heart disease** | I21.1; I21.2; I21.3; I21.4; I21.9; I22.0; I22.1; I22.8; I22.9; I23; I23.0; I23.1; I23.2; I23.3; I23.5; I23.6; I23.8; I24.0; I24.1; I24.8; I24.9; I25.0; I25.1; I25.2; I25.3; I25.4; I25.5; I25.6; I25.8; I25.9; | 131298; 131300; 131302; 131304; 131306 |
| **Stroke** | I60.0; I60.1; I60.2; I60.3; I60.4; I60.5; I60.6; I60.7; I60.8; I60.9; I61.0; I61.1; I61.2; I61.3; I61.4; I61.5; I61.6; I61.8; I61.9; I62.1; I61.9; I63.0; I63.1; I63.2; I63.3; I63.4; I63.5; I63.6; I63.8; I63.9; I64; | 131360; 131362; 131364; 131366; 131368 |
| **Chronic obstructive pulmonary disease** | J43.0; J43.1; J43.2; J43.8; J43.9; J44.0; J44.1; J44.8; J44.9 | 131490; 131492 |
| **Osteoporosis** | M80.0; M80.1; M80.2; M80.4; M80.5; M80.8; M80.9; M81.0; M81.1; M81.2; M81.3; M81.4; M81.5; M81.6; M81.8; M81.9; M82.0; M82.1; M82.8; | 131962; 131964;  131966 |
| **Psoriasis** | L40.0; L40.1; L40.3; L40.4; L40.5; L40.8; L40.9; | 131742 |
| **Systemic Lupus Erythematosus** | M32.0; M32.1; M32.8; M32.9; | 131894 |
| **Rheumatoid Arthritis** | M05.0; M05.1; M05.2; M05.3; M05.8; M05.9;  M06.0; M06.1; M06.2; M06.3; M06.4; M06.8; M06.9; | 131848;  131850; |

ICD: international classification disease.

**Table S4. Baseline characteristics by prevalent valvular heart disease**

|  | **Without prevalent valvular heart disease (*N* =499523)** | **With prevalent valvular heart disease (*N* =2721)** | ***P* value** |
| --- | --- | --- | --- |
| Age (years) | 58.0 (50.0 to 63.0) | 63.0 (57.0 to 66.0) | < 0.001 |
| Female, n (%) | 272125 (54.5) | 1099 (40.4) | < 0.001 |
| Assessment center, n (%) |  |  | 0.04 |
| England | 443218 (88.7) | 2391 (87.9) |  |
| Scotland | 20664 (4.1) | 139 (5.1) |  |
| Wales | 35641 (7.1) | 191 (7.0) |  |
| Race, White ethnicity, n (%) | 469882 (94.1) | 2581 (94.9) | 0.21 |
| Townsend deprivation index | -2.1 (-3.6 to 0.5) | -1.8 (-3.4 to 1.3) | < 0.001 |
| Education, college and above, n (%) | 160373 (32.1) | 670 (24.6) | < 0.001 |
| Current smoker, n (%) | 52711 (10.6) | 234 (8.6) | < 0.001 |
| Current drinker, n (%) | 457795 (91.6) | 2337 (85.9) | < 0.001 |
| Healthy diet score, n (%) |  |  | 0.023 |
| 0-1 | 65952 (13.2) | 336 (12.3) |  |
| 2-3 | 315869 (63.2) | 1689 (62.1) |  |
| 4-5 | 111224 (22.3) | 648 (23.8) |  |
| Missing | 6478 (1.3) | 48 (1.8) |  |
| Sleep duration, hours | 7.0 (7.0 to 8.0) | 7.0 (6.0 to 8.0) | < 0.001 |
| Systolic blood pressure, mmHg | 137.8 (126.0 to 147.5) | 137.8 (126.0 to 147.0) | 0.99 |
| Body mass index, kg/m^2^ | 26.8 (24.2 to 29.9) | 27.4 (24.6 to 30.9) | < 0.001 |
| C-reactive protein, mg/L | 1.5 (0.7 to 2.6) | 2.0 (0.9 to 3.4) | < 0.001 |
| TC, mmol/L | 5.7 (5.0 to 6.4) | 5.1 (4.3 to 5.7) | < 0.001 |
| TG, mmol/L | 1.6 (1.1 to 2.1) | 1.7 (1.1 to 2.1) | < 0.001 |
| HDL-C, mmol/L | 1.4 (1.2 to 1.6) | 1.3 (1.1 to 1.5) | < 0.001 |
| LDL-C, mmol/L | 3.6 (3.0 to 4.1) | 3.1 (2.5 to 3.6) | < 0.001 |
| Glycated haemoglobin, mmol/mol | 35.6 (33.0 to 37.6) | 36.1 (33.9 to 39.3) | < 0.001 |
| Estimated GFR, mL/min/1.73 m^2^ | 91.7 (83.6 to 99.3) | 88.6 (74.5 to 94.1) | < 0.001 |
| Atrial fibrillation, n (%) | 7483 (1.5) | 911 (33.5) | < 0.001 |
| Coronary heart disease, n (%) | 18925 (3.8) | 1042 (38.3) | < 0.001 |
| Stroke, n (%) | 7904 (1.6) | 274 (10.1) | < 0.001 |
| Chronic obstructive pulmonary disease, n (%) | 9555 (1.9) | 178 (6.5) | < 0.001 |
| Osteoporosis, n (%) | 10541 (2.1) | 110 (4.0) | < 0.001 |
| Chronic inflammatory disease, n (%) | 17958 (3.6) | 172 (6.3) | < 0.001 |
| Psoriasis, n (%) | 10974 (2.2) | 79 (2.9) | 0.015 |
| Systemic lupus erythematosus, n (%) | 751 (0.2) | 8 (0.3) | 0.09 |
| Rheumatoid arthritis, n (%) | 6681 (1.3) | 88 (3.2) | < 0.001 |
| Antihypertensive medication, n (%) | 112780 (22.6) | 1943 (71.4) | < 0.001 |
| Antidiabetic medication, n (%) | 18922 (3.8) | 241 (8.9) | < 0.001 |
| Lipid-lowering medication, n (%) | 89453 (17.9) | 1491 (54.8) | < 0.001 |
| Antiplatelet medication, n (%) | 68404 (13.7) | 1093 (40.2) | < 0.001 |

Continuous variables are presented as median (interquartile range) owing to non-normal distribution, and categorical variables are presented as n (%).

TC, total cholesterol; TG, triglycerides; LDL-C, low-density lipoprotein cholesterol; HDL-C, high-density lipoprotein cholesterol; GFR, glomerular filtration rate.

**Table S5. Baseline characteristics of included and excluded participants**

|  | **Included**  **(*N* =499523)** | **Excluded**  **(*N* =21277)** | **Lost to follow-up ***  **(*N* =158)** | ***P* value**  **(Included vs. Excluded)** | ***P* value**  **(Included vs. Lost to follow-up)** |
| --- | --- | --- | --- | --- | --- |
| Age (years) | 58.0 (50.0 to 63.0) | 59.0 (51.0 to 64.0) | 61.0 (53.0 to 65.0) | < 0.001 | < 0.001 |
| Female, n (%) | 262837 (54.6) | 10387 (48.8) | 97 (61.4) | < 0.001 | 0.11 |
| Assessment center, n (%) |  |  |  | < 0.001 | 0.032 |
| England | 426565 (88.7) | 19044 (89.5) | 147 (93.0) |  |  |
| Scotland | 19963 (4.2) | 840 (3.9) | 0 (0.0) |  |  |
| Wales | 34439 (7.2) | 1393 (6.5) | 11 (7.0) |  |  |
| Race, White ethnicity, n (%) | 453491 (94.3) | 18972 (89.2) | 131 (82.9) | < 0.001 | < 0.001 |
| Townsend deprivation index | -2.1 (-3.6 to 0.5) | -1.8 (-3.4 to 1.3) | -2.1 (-3.4 to 0.8) | < 0.001 | 0.68 |
| Education, college and above, n (%) | 156052 (32.4) | 4991 (23.5) | 45 (28.5) | < 0.001 | < 0.001 |
| Current smoker, n (%) | 50658 (10.5) | 2287 (10.7) | 17 (10.8) | < 0.001 | < 0.001 |
| Current drinker, n (%) | 441738 (91.8) | 18394 (86.5) | 128 (81.0) | < 0.001 | < 0.001 |
| Healthy diet score, n (%) |  |  |  | < 0.001 | < 0.001 |
| 0-1 | 63623 (13.2) | 2665 (12.5) | 25 (15.8) |  |  |
| 2-3 | 304923 (63.4) | 12635 (59.4) | 80 (50.6) |  |  |
| 4-5 | 107248 (22.3) | 4624 (21.7) | 35 (22.2) |  |  |
| Missing | 5173 (1.1) | 1353 (6.4) | 18 (11.4) |  |  |
| Sleep duration, hours | 7.0 (7.0 to 8.0) | 7.0 (7.0 to 8.0) | 7.0 (6.0 to 8.0) | < 0.001 | 0.62 |
| Systolic blood pressure, mmHg | 137.8 (125.5 to 148.0) | 137.8 (127.0 to 146.5) | 137.8 (129.5 to 150.0) | 0.004 | 0.017 |
| Body mass index, kg/m^2^ | 26.8 (24.2 to 29.9) | 27.1 (24.3 to 29.5) | 27.4 (24.4 to 28.7) | 0.73 | 0.79 |
| C-reactive protein, mg/L | 1.4 (0.7 to 2.6) | 1.9 (0.8 to 2.6) | 2.4 (1.0 to 2.6) | < 0.001 | 0.003 |
| TC, mmol/L | 5.7 (5.0 to 6.4) | 5.7 (4.8 to 6.1) | 5.7 (5.0 to 6.1) | < 0.001 | 0.45 |
| TG, mmol/L | 1.6 (1.1 to 2.1) | 1.7 (1.1 to 2.0) | 1.7 (1.2 to 1.9) | < 0.001 | 0.47 |
| HDL-C, mmol/L | 1.4 (1.2 to 1.6) | 1.4 (1.2 to 1.5) | 1.4 (1.3 to 1.5) | < 0.001 | 0.44 |
| LDL-C, mmol/L | 3.6 (3.0 to 4.1) | 3.6 (2.9 to 3.9) | 3.6 (3.0 to 3.9) | < 0.001 | 0.55 |
| Glycated haemoglobin, mmol/mol | 35.5 (33.0 to 37.6) | 36.1 (33.8 to 37.9) | 36.1 (35.3 to 37.8) | < 0.001 | < 0.001 |
| Estimated GFR, mL/min/1.73 m^2^ | 91.8 (83.6 to 99.4) | 90.6 (82.8 to 96.9) | 90.6 (87.0 to 96.4) | < 0.001 | 0.08 |
| Atrial fibrillation, n (%) | 6507 (1.4) | 1887 (8.9) | 2 (1.3) | < 0.001 | 1.00 |
| Coronary heart disease, n (%) | 16915 (3.5) | 3052 (14.3) | 7 (4.4) | < 0.001 | 0.68 |
| Stroke, n (%) | 7443 (1.5) | 735 (3.5) | 3 (1.9) | < 0.001 | 0.97 |
| Chronic obstructive pulmonary disease, n (%) | 9062 (1.9) | 671 (3.2) | 3 (1.9) | < 0.001 | 1.00 |
| Osteoporosis, n (%) | 10130 (2.1) | 521 (2.4) | 3 (1.9) | < 0.001 | 1.00 |
| Chronic inflammatory disease, n (%) | 17390 (3.6) | 740 (3.5) | 6 (3.8) | 0.30 | 1.00 |
| Psoriasis, n (%) | 10630 (2.2) | 423 (2.0) | 1 (0.6) | 0.033 | 0.28 |
| Systemic lupus erythematosus, n (%) | 727 (0.2) | 32 (0.2) | 0 (0.0) | 1.00 | 1.00 |
| Rheumatoid arthritis, n (%) | 6467 (1.3) | 302 (1.4) | 5 (3.2) | 0.37 | 0.10 |
| Antihypertensive medication, n (%) | 107491 (22.3) | 7232 (34.0) | 47 (29.7) | < 0.001 | 0.033 |
| Antidiabetic medication, n (%) | 18093 (3.8) | 1070 (5.0) | 7 (4.4) | < 0.001 | 0.82 |
| Lipid-lowering medication, n (%) | 85586 (17.8) | 5358 (25.2) | 40 (25.3) | < 0.001 | 0.018 |
| Antiplatelet medication, n (%) | 65233 (13.6) | 4264 (20.0) | 21 (13.3) | < 0.001 | 1.00 |

Continuous variables are presented as median (interquartile range) owing to non-normal distribution, and categorical variables are presented as n (%).

TC, total cholesterol; TG, triglycerides; LDL-C, low-density lipoprotein cholesterol; HDL-C, high-density lipoprotein cholesterol; GFR, glomerular filtration rate.

***** Lost to follow-up is defined as the National Health Service or UK Biobank system classifying an individual as lost to follow-up at a certain point in time. However, future health records remain accessible, and specific outcome events are still captured.

**Table S6. Drug Classification and Data Coding**

| **Drug classes** | **Data field ID** |
| --- | --- |
| **Antihypertensive medication** | **20003; 6177; 6153** |
| capozide 50mg tablets x28; lisinopril ; carace 2.5mg tablet; zestril 2.5mg tablet; quinapril; accuretic tablet; captopril; acepril 12.5mg tablet; capoten 12.5mg tablet; captopril+hydrochlorothiazide 25mg/12.5mg tablet; innovace 2.5mg tablet; innozide tablet; enalapril maleate+hydrochlorothiazide 20mg/12.5mg tablet; coversyl 2mg tablet; ramipril; staril 10mg tablet; cilazapril; vascace 250micrograms tablet; trandolapril; gopten 500 micrograms capsule; odrik 500micrograms capsule; zestoretic 10 tablet; carace 10 plus tablet; lisinopril+hydrochlorothiazide 10mg/12.5mg tablet; indapamide; amlodipine; accupro 5mg tablet; enalapril; fosinopril; perindopril; felodipine; losartan; cozaar half strength 25mg tablet; perdix 7.5mg tablet; valsartan; diovan 40mg capsule; ecopace 12.5mg tablet; kaplon 12.5mg tablet; losartan potassium+hydrochlorothiazide 10mg/12.5mg tablet; cozaar-comp 50mg/12.5mg tablet; irbesartan; aprovel 75mg tablet; tarka 2mg/180mg m/r capsule; trandolapril+verapamil hydrochloride; candesartan cilexetil; amias 2mg tablet; imidapril hydrochloride; tanatril 5mg tablet; felodipine+ramipril; triapin mite 2.5mg/2.5mg tablet; telmisartan; hyteneze 12.5 tablet; tensopril 12.5mg tablet; capto-co 25mg/12.5mg tablet; pralenal 2.5mg tablet; eprosartan; micardis 20mg tablet; irbesartan+hydrochlorothiazide 150mg/12.5mg tablet; coaprovel 150mg/12.5mg tablet; cozaar 25mg tablet; perindopril+indapamide; coversyl plus 4mg/1.25mg tablet; telmisartan+hydrochlorothiazide 40mg/12.5mg tablet; tritace 1.25mg tablet; caralpha 10/12.5mg tablet; Olmesartan; olmetec 10mg tablet; lopace 2.5mg capsule; ranace 1.25mg capsule; lisicostad hct 10/12.5mg tablet; valsartan+hydrochlorothiazide 80mg/12.5mg tablet; co-diovan 80mg/12.5mg tablet; centyl 2.5mg tablet; urizide 5mg tablet; enduron 5mg tablet; esidrex k tablet; hygroton k tablet combination pack; navidrex-k tablet; serpasil-esidrex tablet; sotalol hydrochloride+hydrochlorothiazide 80mg/12.5mg tablet; metoprolol tartrate+hydrochlorothiazide 100mg/12.5mg tablet; acebutolol+hydrochlorothiazide 200mg/12.5mg tablet; methyldopa+hydrochlorothiazide 250mg/15mg tablet; quinalapril+hydrochlorothiazide 10mg/12.5mg tablet; bisoprolol fumarate+hydrochlorothiazide 10mg/6.25mg tablet; hydroflumethiazide; hydrenox 50mg tablet; mefruside; baycaron 25mg tablet; metolazone; xuret 500micrograms tablet; xipamide; diurexan 20mg tablet; aprinox 2.5mg tablet; berkozide 2.5mg tablet; neo-naclex 5mg tablet; chlorothiazide; saluric 500mg tablet; hygroton 50mg tablet; cyclopenthiazide; navidrex 500mcg tablet; hydrochlorothiazide; esidrex 25mg tablet; hydrosaluric 25mg tablet; amiloride hcl+cyclopenthiazide 2.5mg/250micrograms tablet; centyl k m/r tablet; neo-naclex k m/r tablet; neo-bendromax 2.5mg tablet; nindaxa 2.5mg tablet; chlortalidone; natramid 2.5mg tablet; opumide 2.5mg tablet; diltiazem hcl+hydrochlorothiazide 150mg/12.5mg m/r capsule; natrilix sr 1.5mg m/r tablet; triamterene+chlortalidone 50mg/50mg tablet; atenolol+chlortalidone; diuril 250mg/5ml oral suspension; Bendroflumethiazide; bendroflumethiazide+potassium 2.5mg/7.7mmol m/r tablet; nadolol+bendroflumethiazide 40mg/5mg tablet; timolol maleate+bendroflumethiazide 10mg/2.5mg tablet; atenolol+Bendroflumethiazide; brinaldix k tablet; diatensec 50mg tablet; laractone 25mg tablet; normetic tablet; synuretic tablet; vasetic co-amilozide 5/50mg tablet; pindolol; pindolol+clopamide 10mg/5mg tablet; timolol maleate+co-amilozide 10mg/2.5mg/25mg tablet; aridil 2.5mg/20mg tablet; spiro-co 25mg tablet; midamor 5mg tablet; berkamil 5mg tablet; spiroctan-m 200mg/10ml injection; spironolactone; aldactone 25mg tablet; lasix 20mg tablet; bumetanide; burinex 1mg tablet; spirospare 25mg tablet; spiretic 25mg tablet; spiroctan 25mg tablet; spirolone 25mg tablet; triamterene+benzthiazide 50mg/25mg capsule; triam-co tablet; triamterene+chlorthalidone 50mg/50mg tablet; triamterene+frusemide 50mg/40mg tablet; lasoride tablet; delvas tablet; navispare tablet; amilmaxco 5/50 tablet; burinex a tablet; triamaxco tablet; triamterene; dytac 50mg capsule; aldactide 25 tablet; amil-co tablet; dyazide tablet; dytide capsule; frumil tablet; kalspare tablet; moduret 25 tablet; fru-co tablet; moduretic tablet; amiloride hydrochloride+bumetanide 5mg/1mg tablet; burinex k m/r tablet; diumide-k continus m/r tablet; lasikal m/r tablet; bumetanide+potassium 500micrograms/7.7mmol m/r tablet; diuretic; amiloride; furosemide; amipramizide; zida-co 5mg/50mg tablet; co-triamterzide; co-amilozide; co-flumactone; co-amilofruse; amilamont 5mg/ml s/f oral solution; frusemek 5mg/40mg tablet; atenolol+co-amilozide; angiotensin ii receptor antagonist+diuretic; froop co 5mg/40mg tablet; komil 5/40 tablet; triamterene+furosemide 50mg/40mg tablet; furosemide+potassium 20mg/10mmol m/r tablet; inspra 25mg tablet; spiroprop tablet; metoros 95mg tablet; bedranol 10mg tablet; totamol 25mg tablet; arbralene 50mg tablet; kerlone 20mg tablet; trandate 50mg tablet; betaloc 50mg tablet; mepranix 50mg tablet; metoprolol; artrate+chlorthalidone 100mg/12.5mg tablet; secadrex tablet; sotazide tablet; penbutolol sulphate+frusemide 40mg/20mg tablet; tenoret 50 tablet; tenoretic tablet; tolerzide tablet; viskaldix tablet; timolol maleate+bendrofluazide 10mg/2.5mg tablet; timolol maleate+bendrofluazide 20mg/5mg tablet; atenixco 50mg/12.5mg tablet; beta-adalat capsule; tenif capsule; co-betaloc tablet; inderetic capsule; inderex capsule; kalten capsule; lopresoretic tablet; moducren tablet; prestim tablet; atenolol+nifedipine 50mg/20mg m/r capsule; monocor 5mg tablet; emcor 10mg tablet; celectol 200mg tablet; adalat 5mg capsule; monozide 10 tablet; antipressan 25mg tablet; angilol 10mg tablet; cardinol 10mg tablet; acebutolol; atenolol; tenormin 25 tablet; vasaten 50mg tablet; apsolol 10mg tablet; propanix 10mg tablet; betadur cr 160mg m/r capsule; beta-prograne 160mg m/r capsule; berkolol 10mg tablet; half-betadur cr 80mg m/r capsule; half-inderal la 80mg m/r capsule; half beta-prograne 80mg m/r capsule; inderal 10mg tablet; concordin 5mg tablet; betaxolol; bisoprolol; celiprolol; metoprolol; labetalol; penbutolol; sotalol; timolol; adalate 10mg capsule; avlocardyl retard 160mg m/r capsule; carvedilol; totaretic 50mg/12.5mg tablet; sloprolol 80mg m/r capsule; probeta la 160mg m/r capsule; lopranol la 160mg m/r capsule; atenix 25mg tablet; co-tenidone; atenolol+chlorthalidone; atenolol+bendrofluazide; tenben capsule; half propanix la 80mg m/r capsule; half propatard la 80mg m/r capsule; nebilet 5mg tablet; eucardic 3.125 tablet; dorzolamide+timolol; cardicor 1.25mg tablet; syprol 5mg/5ml oral solution; soloc 5mg tablet; tensomex 100mg tablet; bipranix 5mg tablet; latanoprost+timolol; rapranol sr 80mg m/r capsule; vivacor 5mg tablet  calcicard 60mg tablet; lidoflazine; clinium 120mg tablet; vasad 5mg capsule; synadrin 60mg tablet; veractil 25mg tablet; nifedipine; calcilat 10mg capsule; angiopine 5mg capsule; nifensar xl 20mg m/r tablet; coracten sr 10mg m/r capsule; tildiem 60mg m/r tablet; britiazim 60mg m/r tablet; angiozem 60mg m/r tablet; adizem-60 m/r tablet; cardene 20mg capsule; isradipine; istin 5mg tablet; lacidipine; motens 2mg tablet; half securon sr 120mg m/r tablet; securon 40mg tablet; geangin 40mg tablet; berkatens 40mg tablet; cordilox 40mg tablet; parmid 10mg tablet; nimotop 30mg tablet; nimodipine; nicardipine; univer 120mg m/r capsule; verapamil; nifelease 20mg m/r tablet; slozem 120mg m/r capsule; calanif 5mg capsule; angitil sr 90 m/r capsule; adipine mr 10 m/r tablet; kentiazem 60mg m/r capsule; unipine xl 30mg m/r tablet; adizem-xl plus m/r capsule; cardilate mr 10mg m/r tablet; tensipine mr 10 m/r tablet; plendil 2.5mg m/r tablet; nisoldipine; syscor mr 10mg m/r tablet; fortipine la40 m/r tablet; slofedipine 20mg m/r tablet; nifedotard 20mr m/r tablet; verapress mr 240 m/r tablet; viazem xl 120mg m/r capsule; genalat retard 10mg m/r tablet; lercanidipine; zanidip 10mg tablet; mibefradil; calazem 60mg m/r tablet; optil 60mg m/r tablet; dilcardia sr 60mg m/r capsule; nifedipress mr 10 m/r tablet; nivaten retard 10mg m/r tablet; coroday mr 20mg m/r tablet; zemtard 120 xl m/r capsule; ethimil mr 240 m/r tablet; vertab sr 240 m/r tablet; nifopress retard 20mg m/r tablet; zildil sr 60mg m/r capsule; calchan mr 10mg m/r tablet; zemret 180 xl m/r capsule; bi-carzem sr 60mg m/r capsule; horizem sr 90mg m/r capsule; zolvera 40mg/5ml oral solution; disogram sr 60mg m/r capsule; ranvera mr 240mg m/r tablet; cabren 2.5mg m/r tablet; vera-til sr 120mg m/r tablet; kentipine mr 10mg m/r tablet; felotens xl 5mg m/r tablet; felogen xl 5mg m/r tablet; hypolar retard 10mg m/r tablet; valni 20 retard 20mg m/r tablet; cardioplen xl 5mg m/r tablet; amlostin 5mg tablet; neofel xl 5mg m/r tablet; parmid xl 5mg m/r tablet.  1140851692; 1140860696; 1140860706; 1140860714; 1140860728; 1140860736; 1140860750; 1140860752; 1140860758; 1140860764; 1140860776; 1140860784; 1140860790; 1140860802; 1140860806; 1140860878; 1140860882; 1140860892; 1140860904; 1140860912; 1140860918; 1140864618; 1140864910; 1140864952; 1140866078; 1140879802; 1140881706; 1140888552; 1140888556; 1140888560; 1140888646; 1140916356; 1140916362; 1140923718; 1141145660; 1141145668; 1141150328; 1141150560; 1141151016; 1141151018; 1141152998; 1141153006; 1141153316; 1141153328; 1141156836; 1141156846; 1141164148; 1141164154; 1141165470; 1141165476; 1141166006; 1141167758; 1141167822; 1141170544; 1141170870; 1141171336; 1141172492; 1141172682; 1141172686; 1141179974; 1141180592; 1141180598; 1141187788; 1141188408; 1141190934; 1141193282; 1141193346; 1141199940; 1141200698; 1141200726; 1141201038; 1141201040; 1140851332; 1140851336; 1140851338; 1140851362; 1140851364; 1140851368; 1140851660; 1140860332; 1140860404; 1140860422; 1140860562; 1140860738; 1140864950; 1140866072; 1140866074; 140866084; 1140866086; 1140866092; 1140866096; 1140866108; 1140866110; 1140866128; 1140866132; 1140866136; 1140866138; 1140866140; 1140866146; 1140866156; 1140866158; 1140866162; 1140866164; 1140866168; 1140866422; 1140866440; 1140866446; 1140888918; 1140888922; 1140909706; 1140916870; 1140917068; 1140926778; 1141146378; 1141180772; 1141180778; 1141188636; 1141194794; 1141194800; 1141194804; 1141194808; 1141194810; 1140851360; 1140851418; 1140851420; 1140851428; 1140851430; 1140851436; 1140860292; 1140860322; 1140860336;1140864550; 1140864574; 1140866220; 1140866226; 1140866232; 1140866236; 1140866244; 1140866248; 1140866280; 1140866282; 1140866306; 1140866308; 1140866312; 1140866318; 1140866324; 1140866328; 1140866330; 1140866332; 1140866334; 1140866340; 1140866352; 1140866354; 1140866356; 1140866360; 1140866388; 1140866390; 1140866396; 1140866400; 1140866402; 1140866404; 1140866406; 1140866410; 1140866416; 1140866418; 1140866420; 1140866426; 1140866438; 1140866442; 1140866444; 1140866448; 1140881894; 1140888512; 1140909708; 1140909722; 1140922324; 1140923272; 1140923276; 1140923282; 1140923402; 1140927174; 1140928624; 1141146128; 1141150898; 1141167108; 1141181520; 1141195254; 1141195258; 1141201250; 1140851508; 1140851522; 1140851556; 1140860172; 1140860180; 1140860232; 1140860250; 1140860266; 1140860278; 1140860308; 1140860314; 1140860318; 1140860320; 1140860324; 1140860328; 1140860330; 1140860338; 1140860340; 1140860342; 1140860348; 1140860356; 1140860358; 1140860386; 1140860394; 1140860396; 1140860398; 1140860402; 1140860406; 1140860410; 1140860426; 1140860434; 1140860492; 1140860498; 1140861090; 1140864176; 1140864410; 1140866704; 1140866712; 1140866724; 1140866738; 1140866756; 1140866758; 1140866764; 1140866766; 1140866778; 1140866782; 1140866784; 1140866798; 1140866800; 1140866802; 1140866804; 1140867734; 1140879758; 1140879760; 1140879762; 1140879818; 1140879824; 1140879834; 1140879854; 1140879866; 1140881702; 1140881722; 1140909368; 1140916628; 1140916730; 1140916868; 1140917076; 1140922930; 1140923336; 1141146124; 1141146126; 1141146184; 1141152076; 1141156754; 1141164280; 1141168498; 1141169516; 1141171152; 1141172742; 1141182904; 1141182968; 1141184324; 1141184722; 1141187048; 1141187780; 1140851730; 1140851784; 1140851786; 1140851790; 1140851800; 1140855976; 1140861088; 1140861106; 1140861110; 1140861114; 1140861120; 1140861128; 1140861130; 1140861136; 1140861138; 1140861176; 1140861190; 1140861202; 1140861276; 1140861282; 1140866460; 1140866466; 1140866484; 1140866546; 1140866554; 1140868036; 1140872472; 1140872568; 1140879810; 1140881692; 1140888510; 1140911088; 1140911698; 1140916930; 1140917428; 1140923572; 1140923618; 1140926188; 1140926780; 1140927934; 1140927940; 1140928212; 1140928226; 1140928234; 1141145870; 1141150500; 1141150538; 1141150926; 1141151474; 1141152600; 1141153026; 1141153032; 1141153394; 1141153454; 1141156656; 1141157136; 1141157140; 1141162546; 1141166752; 1141167832; 1141169096; 1141169710; 1141169730; 1141171804; 1141173766; 1141174684; 1141175224; 1141180238; 1141184390; 1141185444; 1141187056; 1141187094; 1141187774; 1141187962; 1141188152; 1141188576; 1141188936; 1141190548; 1141199858; 1141200400; 1141200782; 1141201814 | |
| **Antidiabetic medication** | **20003; 6177; 6153** |
| Glibornuride; daonil 5mg tablet; glutril 25mg tablet; semi-daonil 2.5mg tablet; glymidine; euglucon 2.5mg tablet; gondafon 500mg tablet ; malix 2.5mg tablet ; pramidex 500mg tablet; diabetamide 2.5mg tablet; glipizide; gliclazide; glibenese 5mg tablet; diamicron 80mg tablet; minodiab 2.5mg tablet; insulin product; tolazamide; metformin; tolanase 100mg tablet; glimepiride; tolbutamide; troglitazone; glyconon 500mg tablet; romozin 200mg tablet ; rastinon 500mg tablet ; amaryl 1mg tablet ; glucophage 500mg tablet; pioglitazone; orabet 500mg tablet; actos 15mg tablet; chlorpropamide; rosiglitazone; diabinese 100mg tablet; avandia 4mg tablet; glymese 250mg tablet; rosiglitazone 1mg / metformin 500mg tablet; glibenclamide; avandamet 1mg / 500mg tablet  1140857494; 1140874724; 1140857496; 1140874726;1140857500;1140874728; 1140857502; 1140874732; 1140857506; 1140874736; 1140874646; 1140874744; 1140874650; 1140874746; 1140874652; 1140883066; 1140874664; 1140884600; 1140874666; 1141152590; 1140874674; 1141153254; 1140874678; 1141153262; 1140874680; 1141156984; 1140874686; 1141171646; 1140874690; 1141171652; 1140874706; 1141177600; 1140874712; 1141177606; 1140874716; 1141189090; 1140874718; 1141189094; | |
| **Lipid-lowering medication** | **20003; 6177; 6153** |
| Lipid Lowering Drug; Atorvastatin; Velastatin; Rosuvastatin; Simvastatin; Fluvastatin; pravastatin;Eptastatin; lipostat 10mg tablet; Atromid-s 500mg capsule; Modalim 100mg Tablet; crestor 10mg tablet; Bezafibrate; Fenofibrate; Bezafibrate product; Clofibrate; Gemfibrozil; Gemfibrozil product; bezalip; bezalip-mono 400mg m/r tablet; questran 4g/sachet powder; cholestyramine+aspartame 4g/sachet powder; lopid 300 capsule; colestipol; synvinolin; Acipimox; Nicotinic Acid Product; probucol;nicofuranose; lurselle 250mg tablet; colestid 5g/sachet granules; olbetam 250mg capsule; cholestyramine; colestyramine; cholestyramine product;  1140861922; 1141146234; 1140910654; 1141192410; 1140861958; 1140888594; 1140888648; 1140861970; 1140910632; 140861946;1140862028; 1141192414; 1140861924; 1140861954; 1141157260; 1140861944; 1140861856; 1141157262; 1140861926; 1140861928; 1140861936; 1140861942; 140861858;1140888590;1140910652;1140861868;1140861892;1140861876; 1140861866; 1140861848; 1140861878;1140861894; 1140865576;1140909780; 1141157416; | |
| **Antiplatelet medication** | **20003; 6154** |
| Aspirin; antiplatelet drug; aspirin; aspirin 75mg tablet; nu-seals aspirin 75mg e/c tablet; aspirin+methocarbamol 325mg/400mg tablet; aspirin+metoclopramide 325mg/5mg effervescent tablet; aspirin+glycine 500mg/133mg dispersible tablet; aspirin+codeine 300mg/8mg tablet; aspirin+codeine; aspirin+papaveretum 500mg/7.71mg dispersible tablet; isosorbide mononitrate+aspirin; dipyridamole+aspirin; disprin cv 100mg m/r tablet; disprin direct dispersible tablet; acetylsalicylic acid; anadin tablet; aspro clear maximum strength soluble tablet; codis dispersible tablet; alka-seltzer tablet; norgesic tablet; persantin 25mg tablet; cerebrovase 25mg tablet; angettes 75mg tablet; aspav dispersible tablet; aspirin+cyclizine hydrochloride 500mg/25mg tablet; sulfinpyrazone; asasantin retard m/r capsule; clopidogrel; plavix 75mg tablet; platet 100mg effervescent tablet；ticlopidine; dipyridamole; pentoxifylline;  1140861776; 1140868226;1140861806;1140864860;1140868282;1140872040;1140882190;1140882268;1140882392;1141163138;1141164044;1141167844;1140861808;1140882192; 1140909772; 1140911754; 1140909480; 1140856336; 1140917114; 1140856412; 1140861780; 1140861804; 1140868258; 1140882108; 1140909890; 1141167848; 1141168318; 1141168322; 1140861800; 1141163324; 1140861778; 1140909712 | |

**Table S7. Age- and sex-specific associations of physical frailty status with risk of incident AS and AS-related events**

|  | **Robust** | **Prefrail** | **Frail** | ***P* for trend *** | ***P* for interaction** |
| --- | --- | --- | --- | --- | --- |
| **AS** |  |  |  |  |  |
| Age < 60y | Reference | 1.33 (1.17-1.51) | 1.80 (1.46-2.21) | < 0.001 | 0.016 |
| Age ≥ 60y | Reference | 1.28 (1.20-1.37) | 1.61 (1.43-1.80) | < 0.001 |  |
| Women | Reference | 1.35 (1.21-1.49) | 1.71 (1.46-2.00) | < 0.001 | 0.051 |
| Men | Reference | 1.27 (1.18-1.37) | 1.61 (1.41-1.85) | < 0.001 |  |
| **AS-related events** |  |  |  |  |  |
| Age < 60y | Reference | 1.27 (1.05-1.53) | 1.87 (1.35-2.58) | < 0.001 | 0.17 |
| Age ≥ 60y | Reference | 1.32 (1.18-1.46) | 1.44 (1.18-1.74) | < 0.001 |  |
| Women | Reference | 1.44 (1.21-1.71) | 1.72 (1.32-2.25) | < 0.001 | 0.031 |
| Men | Reference | 1.26 (1.13-1.41) | 1.43 (1.15-1.77) | < 0.001 |  |

AS, aortic valve stenosis.

Models were adjusted for age, sex (only in age-specific analyses), race, UK Biobank assessment center, Townsend deprivation index, education, smoking status, alcohol consumption status, healthy diet score, sleep duration, body mass index, systolic blood pressure, glycated haemoglobin, total cholesterol, triglycerides, low-density lipoprotein cholesterol, high-density lipoprotein cholesterol, estimated glomerular filtration rate, C-reactive protein, coronary heart disease, atrial fibrillation, stroke, chronic obstructive pulmonary disease, osteoporosis, chronic inflammatory disease, antihypertensive medication, antidiabetic medication, Lipid-lowering medication, and Antiplatelet medication.

^*^*P* for trend was calculated by using median physical frailty score value (0, 1 and 3) of each frailty status.

**Table S8. Age- and sex-specific associations of individual components of physical frailty with risk of incident AS and AS-related events**

|  | **Weight loss** | **Exhaustion** | **Low physical activity** | **Slow gait speed** | **Low grip strength** |
| --- | --- | --- | --- | --- | --- |
| **AS** |  |  |  |  |  |
| Age < 60y | 1.30 (1.14-1.50) | 1.27 (1.10-1.47) | 0.93 (0.79-1.09) | 1.43 (1.20-1.69) | 1.17 (1.03-1.33) |
| Age ≥ 60y | 1.29 (1.19-1.40) | 1.11 (1.01-1.21) | 1.07 (0.98-1.17) | 1.28 (1.17-1.39) | 1.18 (1.10-1.25) |
| *P* for interaction | 0.78 | 0.007 | 0.86 | 0.005 | 0.41 |
| Women | 1.33 (1.19-1.48) | 1.12 (1.00-1.26) | 1.04 (0.92-1.17) | 1.41 (1.25-1.59) | 1.14 (1.04-1.25) |
| Men | 1.27 (1.17-1.39) | 1.18 (1.07-1.31) | 1.03 (0.93-1.15) | 1.23 (1.11-1.36) | 1.20 (1.11-1.29) |
| *P* for interaction | 0.47 | 1.00 | 0.22 | 0.002 | 0.51 |
| **AS-related events** |  |  |  |  |  |
| Age < 60y | 1.26 (1.02-1.56) | 1.24 (0.99-1.56) | 1.08 (0.84-1.37) | 1.29 (0.98-1.70) | 1.14 (0.94-1.39) |
| Age ≥ 60y | 1.20 (1.06-1.36) | 1.04 (0.89-1.21) | 1.10 (0.95-1.27) | 1.13 (0.98-1.30) | 1.25 (1.13-1.37) |
| *P* for interaction | 0.68 | 0.18 | 0.73 | 0.27 | 0.44 |
| Women | 1.21 (1.01-1.45) | 1.12 (0.92-1.36) | 1.02 (0.83-1.25) | 1.33 (1.09-1.63) | 1.18 (1.02-1.38) |
| Men | 1.21 (1.06-1.38) | 1.08 (0.92-1.27) | 1.14 (0.97-1.33) | 1.06 (0.91-1.25) | 1.25 (1.12-1.39) |
| *P* for interaction | 0.74 | 0.44 | 0.81 | 0.008 | 0.54 |

AS, aortic valve stenosis.

Models were adjusted for age, sex (only in age-specific analyses), race, UK Biobank assessment center, Townsend deprivation index, education, smoking status, alcohol consumption status, healthy diet score, sleep duration, body mass index, systolic blood pressure, glycated haemoglobin, total cholesterol, triglycerides, low-density lipoprotein cholesterol, high-density lipoprotein cholesterol, estimated glomerular filtration rate, C-reactive protein, coronary heart disease, atrial fibrillation, stroke, chronic obstructive pulmonary disease, osteoporosis, chronic inflammatory disease, antihypertensive medication, antidiabetic medication, Lipid-lowering medication, Antiplatelet medication, and five frailty components (mutual adjustment).

**Table S9. Sensitivity analysis by excluding events occurred within the first two years of follow-up**

|  |  | **AS** | **AS-related events** |
| --- | --- | --- | --- |
| **Physical frailty status** | Robust | Reference | Reference |
|  | Prefrail | 1.29 (1.21-1.37) | 1.28 (1.17-1.41) |
|  | Frail | 1.64 (1.47-1.81) | 1.51 (1.27-1.79) |
|  | *P* for trend | < 0.001 | < 0.001 |
| **Individual components of physical frailty** | Weight loss | 1.31 (1.23-1.41) | 1.25 (1.12-1.39) |
|  | Exhaustion | 1.14 (1.05-1.24) | 1.08 (0.95-1.24) |
|  | Low physical activity | 1.03 (0.95-1.12) | 1.09 (0.95-1.24) |
|  | Slow gait speed | 1.29 (1.19-1.39) | 1.14 (1.00-1.30) |
|  | Low grip strength | 1.17 (1.11-1.24) | 1.21 (1.11-1.33) |

AS, aortic valve stenosis.

Models were adjusted for age, sex, race, UK Biobank assessment center, Townsend deprivation index, education, smoking status, alcohol consumption status, healthy diet score, sleep duration, body mass index, systolic blood pressure, glycated haemoglobin, total cholesterol, triglycerides, low-density lipoprotein cholesterol, high-density lipoprotein cholesterol, estimated glomerular filtration rate, C-reactive protein, coronary heart disease, atrial fibrillation, stroke, chronic obstructive pulmonary disease, osteoporosis, chronic inflammatory disease, antihypertensive medication, antidiabetic medication, Lipid-lowering medication, and Antiplatelet medication.

**Table S10. Sensitivity analysis by excluding** **participants with atrial fibrillation, coronary heart disease, or stroke at baseline**

|  |  | **AS** | **AS-related events** |
| --- | --- | --- | --- |
| **Physical frailty status** | Robust | Reference | Reference |
|  | Prefrail | 1.24 (1.16-1.32) | 1.27 (1.15-1.40) |
|  | Frail | 1.65 (1.47-1.85) | 1.50 (1.24-1.82) |
|  | *P* for trend | < 0.001 | < 0.001 |
| **Individual components of physical frailty** | Weight loss | 1.27 (1.18-1.37) | 1.21 (1.07-1.36) |
|  | Exhaustion | 1.14 (1.04-1.24) | 1.07 (0.92-1.23) |
|  | Low physical activity | 1.05 (0.96-1.15) | 1.09 (0.95-1.25) |
|  | Slow gait speed | 1.31 (1.20-1.43) | 1.15 (1.00-1.33) |
|  | Low grip strength | 1.16 (1.09-1.24) | 1.21 (1.10-1.33) |

AS, aortic valve stenosis.

Models were adjusted for age, sex, race, UK Biobank assessment center, Townsend deprivation index, education, smoking status, alcohol consumption status, healthy diet score, sleep duration, body mass index, systolic blood pressure, glycated haemoglobin, total cholesterol, triglycerides, low-density lipoprotein cholesterol, high-density lipoprotein cholesterol, estimated glomerular filtration rate, C-reactive protein, coronary heart disease, atrial fibrillation, stroke, chronic obstructive pulmonary disease, osteoporosis, chronic inflammatory disease, antihypertensive medication, antidiabetic medication, Lipid-lowering medication, and Antiplatelet medication.

**Table S11. Sensitivity analysis by treating death due to other causes as competing risks**

|  |  | **AS** | **AS-related events** |
| --- | --- | --- | --- |
| **Physical frailty status** | Robust | Reference | Reference |
|  | Prefrail | 1.25 (1.17-1.33) | 1.24 (1.13-1.37) |
|  | Frail | 1.40 (1.24-1.58) | 1.37 (1.13-1.65) |
|  | *P* for trend | < 0.001 | < 0.001 |
| **Individual components of physical frailty** | Weight loss | 1.29 (1.19-1.39) | 1.20 (1.07-1.34) |
|  | Exhaustion | 1.11 (1.02-1.21) | 1.07 (0.94-1.23) |
|  | Low physical activity | 0.99 (0.91-1.09) | 1.04 (0.90-1.20) |
|  | Slow gait speed | 1.15 (1.05-1.26) | 1.08 (0.94-1.24) |
|  | Low grip strength | 1.15 (1.08-1.22) | 1.20 (1.10-1.32) |

AS, aortic valve stenosis.

Models were adjusted for age, sex, race, UK Biobank assessment center, Townsend deprivation index, education, smoking status, alcohol consumption status, healthy diet score, sleep duration, body mass index, systolic blood pressure, glycated haemoglobin, total cholesterol, triglycerides, low-density lipoprotein cholesterol, high-density lipoprotein cholesterol, estimated glomerular filtration rate, C-reactive protein, coronary heart disease, atrial fibrillation, stroke, chronic obstructive pulmonary disease, osteoporosis, chronic inflammatory disease, antihypertensive medication, antidiabetic medication, Lipid-lowering medication, and Antiplatelet medication.

**Table S12. Sensitivity analysis by imputing covariates using 30 multiple imputation samples**

|  |  | **AS** | **AS-related events** |
| --- | --- | --- | --- |
| **Physical frailty status** | Robust | Reference | Reference |
|  | Prefrail | 1.30 (1.22-1.38) | 1.31 (1.19-1.43) |
|  | Frail | 1.66 (1.50-1.84) | 1.53 (1.30-1.81) |
|  | *P* for trend | < 0.001 | < 0.001 |
| **Individual components of physical frailty** | Weight loss | 1.30 (1.22-1.39) | 1.22 (1.10-1.36) |
|  | Exhaustion | 1.16 (1.07-1.25) | 1.10 (0.97-1.24) |
|  | Low physical activity | 1.04 (0.96-1.12) | 1.09 (0.96-1.24) |
|  | Slow gait speed | 1.30 (1.20-1.40) | 1.16 (1.02-1.31) |
|  | Low grip strength | 1.18 (1.12-1.25) | 1.23 (1.13-1.34) |

AS, aortic valve stenosis.

Models were adjusted for age, sex, race, UK Biobank assessment center, Townsend deprivation index, education, smoking status, alcohol consumption status, healthy diet score, sleep duration, body mass index, systolic blood pressure, glycated haemoglobin, total cholesterol, triglycerides, low-density lipoprotein cholesterol, high-density lipoprotein cholesterol, estimated glomerular filtration rate, C-reactive protein, coronary heart disease, atrial fibrillation, stroke, chronic obstructive pulmonary disease, osteoporosis, chronic inflammatory disease, antihypertensive medication, antidiabetic medication, Lipid-lowering medication, and Antiplatelet medication.

**Table S13. Associations of polygenic risk score with the risk of incident aortic valve stenosis**

|  | **Model 1** | **Model 2** | **Model 3** |
| --- | --- | --- | --- |
| **PRS categories** |  |  |  |
| Low genetic risk | Reference | Reference | Reference |
| Intermediate genetic risk | 1.16 (1.07-1.25) | 1.15 (1.07-1.24) | 1.15 (1.07-1.24) |
| High genetic risk | 1.51 (1.40-1.62) | 1.51 (1.40-1.62) | 1.49 (1.38-1.60) |
| **PRS, per SD increment*** | 1.21 (1.18-1.24) | 1.21 (1.18-1.24) | 1.19 (1.16-1.22) |

PRS, polygenic risk score; SD, standard deviation.

* SD of PRS was 0.043.

Model 1 adjusted for none.

Model 2 adjusted for age, sex, race, UK Biobank assessment center, Townsend deprivation index, education, smoking status, alcohol consumption status, healthy diet score, and sleep duration.

Model 3 included model 1 plus body mass index, systolic blood pressure, glycated haemoglobin, total cholesterol, triglycerides, low-density lipoprotein cholesterol, high-density lipoprotein cholesterol, estimated glomerular filtration rate, C-reactive protein, coronary heart disease, atrial fibrillation, stroke, chronic obstructive pulmonary disease, osteoporosis, chronic inflammatory disease, antihypertensive medication, antidiabetic medication, lipid-lowering medication, and antiplatelet medication.

**Table S14.** Risk of incident aortic valve stenosis according to physical frailty status within polygenic risk category

|  | **Polygenic risk category** | | | ***P* value for**  **interaction** |
| --- | --- | --- | --- | --- |
|  | **Low genetic risk** | **Intermediate genetic risk** | **High genetic risk** |  |
| **AS** |  |  |  |  |
| Robust | Reference | Reference | Reference | 0.033 |
| Prefrail | 1.21 (1.10-1.35) | 1.32 (1.16-1.51) | 1.41 (1.25-1.59) |  |
| Frail | 1.58 (1.32-1.88) | 1.63 (1.32-2.01) | 1.93 (1.56-2.38) |  |

AS, aortic valve stenosis.

Models adjusted for age, sex, race, UK Biobank assessment center, Townsend deprivation index, education, smoking status, alcohol consumption status, healthy diet score, sleep duration, body mass index, systolic blood pressure, glycated haemoglobin, total cholesterol, triglycerides, low-density lipoprotein cholesterol, high-density lipoprotein cholesterol, estimated glomerular filtration rate, C-reactive protein, coronary heart disease, atrial fibrillation, stroke, chronic obstructive pulmonary disease, osteoporosis, chronic inflammatory disease, antihypertensive medication, antidiabetic medication, lipid-lowering medication, and antiplatelet medication.
